# Landscape of transcription termination in Arabidopsis revealed by single-molecule nascent RNA sequencing

**DOI:** 10.1186/s13059-021-02543-4

**Published:** 2021-11-25

**Authors:** Weipeng Mo, Bo Liu, Hong Zhang, Xianhao Jin, Dongdong Lu, Yiming Yu, Yuelin Liu, Jinbu Jia, Yanping Long, Xian Deng, Xiaofeng Cao, Hongwei Guo, Jixian Zhai

**Affiliations:** 1 Department of Biology, School of Life Sciences, Southern University of Science and Technology, Shenzhen, 518055 China; 2 Institute of Plant and Food Science, Southern University of Science and Technology, Shenzhen, 518055 China; 3 Key Laboratory of Molecular Design for Plant Cell Factory of Guangdong Higher Education Institutes, Southern University of Science and Technology, Shenzhen, 518055 China; 4 State Key Laboratory of Plant Genomics and National Center for Plant Gene Research (Beijing), Institute of Genetics and Developmental Biology, Chinese Academy of Sciences, Beijing, 100101 China

**Keywords:** Transcription termination, Nascent RNA, Nanopore sequencing, Arabidopsis

## Abstract

**Background:**

The dynamic process of transcription termination produces transient RNA intermediates that are difficult to distinguish from each other via short-read sequencing methods.

**Results:**

Here, we use single-molecule nascent RNA sequencing to characterize the various forms of transient RNAs during termination at genome-wide scale in wildtype Arabidopsis and in *atxrn3*, *fpa*, and *met1* mutants. Our data reveal a wide range of termination windows among genes, ranging from ~ 50 nt to over 1000 nt. We also observe efficient termination before downstream tRNA genes, suggesting that chromatin structure around the promoter region of tRNA genes may block pol II elongation. 5′ Cleaved readthrough transcription in *atxrn3* with delayed termination can run into downstream genes to produce normally spliced and polyadenylated mRNAs in the absence of their own transcription initiation. Consistent with previous reports, we also observe long chimeric transcripts with cryptic splicing in *fpa* mutant; but loss of CG DNA methylation has no obvious impact on termination in the *met1* mutant.

**Conclusions:**

Our method is applicable to establish a comprehensive termination landscape in a broad range of species.

**Supplementary Information:**

The online version contains supplementary material available at 10.1186/s13059-021-02543-4.

## Background

Transcription termination is the critical final step in RNA synthesis, during which nascent RNA is released from the complex of RNA Polymerase II (Pol II) and DNA template [1]. Termination is essential to prevent uncontrolled readthrough transcription from invading downstream genes [2, 3]. Decades of extensive research in this area has proposed the allosteric/antiterminator model [4], the torpedo model [5], and later a unified model that combines these two to explain the mechanism of polyadenylation signal (PAS)-dependent termination [1, 3, 6–8]. In mammalian cells, after Pol II passed through the poly(A) site, cleavage and polyadenylation process is initiated, and transcription continues until Pol II disassociated from the DNA template and nascent RNA released [2, 6]. The 3′ end of mature mRNA is determined by the cleavage event, rather than the site where transcription terminates. The cleavage at poly(A) site provides an entry for 5′ → 3′ exonuclease (Xrn2 in human, Rat1 in yeast, AtXRN2/3 in Arabidopsis) to degrade the 3′ cleavage product that is still presumably being synthesized by Pol II [6, 9–11]. The readthrough transcripts accumulate in mutants with a defective cleavage and polyadenylation complex [12, 13] or a loss-of-function exonuclease [9, 14, 15]. The kinetic competition between Pol II and exonuclease promotes termination [1, 2, 16]. As a result, readthrough transcription can continue for up to a few thousand nucleotides (nt) downstream of the poly(A) site [17, 18]. Since the Arabidopsis genome is highly compact, with ~ 38,000 genes concentrated in only ~ 135 Mb DNA sequence [19], termination is essential to prevent the transcriptional collisions between flanking genes. To date, the pattern of Pol II termination in plants remains largely unknown. Thus, we set out to study transcription termination in the model plant Arabidopsis.

The majority of the termination RNA intermediates are transient and rapidly degraded after cleavage, and are therefore difficult to capture and characterize. Several short-read Illumina-based methods have been developed to analyze nascent RNAs [20], but their aims are primarily on the elongating fraction of RNAs before termination, rather than the readthrough and cleaved ones. GRO-seq [21] and PRO-seq [22] performed a run-on with isolated nuclei, which might compromise the termination machinery. NET-seq [23], mNET-seq [24], and POINT-seq [25] require immunoprecipitation of Pol II, which might miss terminating RNAs that are no longer associated with Pol II, such as the cleaved and polyadenylated pre-mRNAs after Pol II passing through the poly(A) site, and cannot distinguish between individual peaks caused by Pol II accumulation and co-transcriptional cleavage [20–24]. TT-seq [17] was indeed able to define transcription termination sites, but due to the limited read length of Illumina sequencing and the RNA fragmentation step during library construction, it also cannot distinguish whether the readthrough transcripts are cleaved or not. Therefore, the field of transcription termination would benefit from a new high-throughput method that can capture and distinguish the various forms of RNA intermediates.

Recent advances in third-generation long-read sequencing technologies, such as PacBio and Nanopore [26, 27], have enabled the detection of full-length mRNAs or cDNAs [28–32]. In particular, the Oxford Nanopore direct RNA sequencing (ONT DRS) can recognize and distinguish various forms of RNA modifications that cause unique changes of current when RNA molecule is passing through the nanopore [26]. The applications of long-read sequencing (LRS) in characterizing full-length nascent RNAs have revealed co-transcriptional splicing kinetics in a variety of species by simultaneously tracking splicing status and position of Pol II elongation on the same RNA molecule, such as nano-COP in human and drosophila [33, 34], LRS of nascent RNA in yeast and mouse [35, 36], POINT-nano in human [25], and recent work from our group in Arabidopsis [37]. Here, we demonstrate the application of single-molecule nascent RNA sequencing in studying transcription termination by simultaneously recording Pol II readthrough distance, cleavage status, and polyadenylation on the same RNA molecule, and revealed the global landscape of transcription termination in Arabidopsis.

## Results

### Single-molecule nascent RNA sequencing captures RNA intermediates during termination

**Fig. 1:**
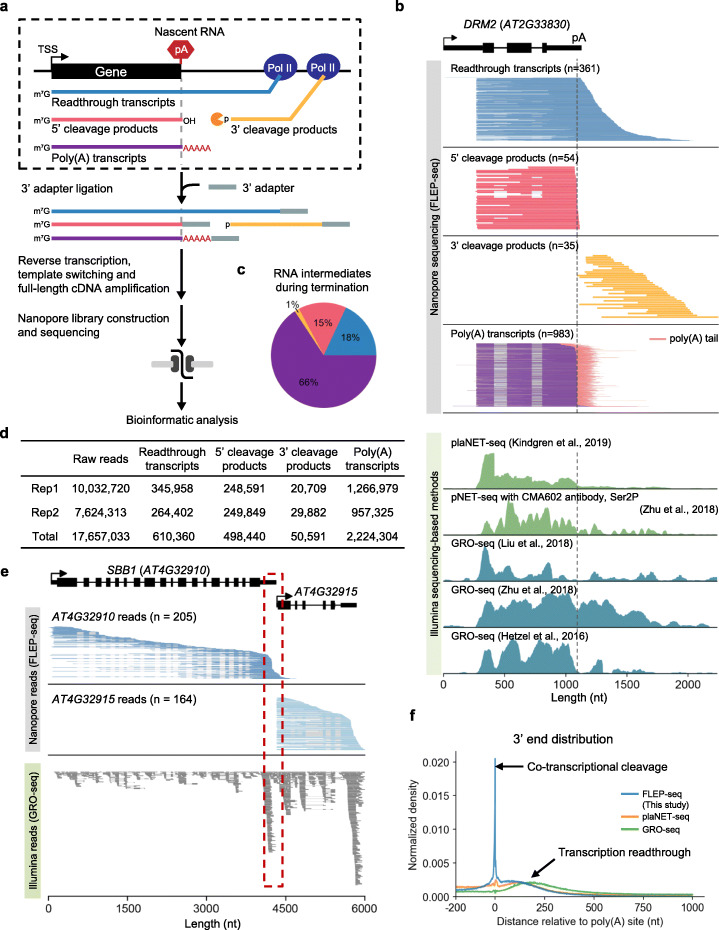
Single-molecule Nascent RNA Sequencing (FLEP-seq) captures various transient RNA intermediates produced during transcription termination. **a** Schematic of the FLEP-seq library preparation and sequencing. A 3′ adapter is ligated to nascent RNA for reverse transcription and full-length cDNA cloning. **b** Example of the RNA intermediates from gene *DRM2*. Upper panel: Nanopore reads are aligned to gene ordered by 3′ end position. The number of individual long reads (*n*) is indicated. Lower panel: coverage of Illumina short-read nascent RNA data from previous studies [39–42]. The gray dash line indicates the poly(A) site (pA). **c** Proportion of various RNA intermediates during termination. Readthrough transcripts (blue), 5′ cleavage products (red), 3′ cleavage products (yellow) and poly(A) transcripts (purple). **d** Sequencing summary for two biological replicates of FLEP-seq libraries in wildtype Col-0. **e** Advantage of Nanopore (upper) over Illumina [40] (lower) in separating reads from closely adjacent genes. The red dashed box highlights the intergenic region. **f** Meta-profile showing the 3′ end distribution of non-poly(A) + nascent RNA detected by the FLEP-seq, plaNET-seq [42] reads, and GRO-seq [40] near poly(A) site

Our group recently developed a single-molecule nascent RNA sequencing method named FLEP-seq (Full-Length Elongating and Polyadenylated RNA sequencing) [38] by using the chromatin-bound nascent RNA to study the coordination between Pol II elongation and splicing [37]. Here, we demonstrate that the comprehensive profile of full-length nascent RNAs in Arabidopsis captured by FLEP-seq can be applied to study transcription termination by characterizing the various forms of RNA intermediate produced during the termination process (Fig. 1a). Compared to previous short-read Illumina-based methods, the long-read Nanopore sequencing of FLEP-seq enables us to distinguish the full-length readthrough RNAs from the 5′ and 3′ cleaved transient RNAs (Fig. 1a), which are generated by the 3′-end processing factors at the poly(A) site [7, 43]. FLEP-seq also captures the nascent polyadenylated mRNAs that are still associated with chromatin, thus allow us to obtain the precise location of poly(A) site for each gene [30, 44] (Additional file 1, Fig. S1a-c). Given the heterogeneity of poly(A) sites at Arabidopsis 3′ UTRs [19, 45], having both the poly(A) site information and the cleavage information in the same library can facilitate our analysis by enabling a more accurate assessment of cleavage events.

The advantage of having the unfractionated long RNAs from FLEP-seq data enabled us to distinguish and categorize the RNA intermediates into four groups: readthrough transcripts, 5′ cleavage products, 3′ cleavage products, and poly(A) transcripts (Fig. 1a; Additional file 1, Fig. S2). Take *DRM2* (*AT2G33830*) as an example (Fig. 1b, upper panel), readthrough transcripts are those that extended downstream of the poly(A) site; 5′ cleavage products are those cleaved at the poly(A) site but not yet polyadenylated; 3′ cleavage products are full-length transcripts with their 5′ ends located downstream of the poly(A) site; and poly(A) transcripts are RNAs with poly(A) tails, and this result is also used to define the position of poly(A) site. Previous short-read based nascent RNA sequencing methods, such as pNET-seq, plaNET-seq, and GRO-seq have been applied in Arabidopsis [39–42] and in other plants including cassava and maize [46], are mostly developed for detecting Pol II-associated elongating RNAs, and can also detect RNA signal downstream of poly(A) site (Fig. 1b, lower panel). In particular, the pNET-seq method can detect nascent RNAs that are associated with various isoforms of Pol II CTD, including total CTD, unphosphorylated, phosphorylation on Ser 2 and Ser 5 [41], and thus may distinguish Pol II at certain transcription step. Compared to these methods, our FLEP-seq method captures a much more comprehensive and smoother pattern of termination and has the unique advantage of distinguishing whether the RNA intermediates are cleaved or not (Fig. 1b). Moreover, in the cases where two neighboring genes are immediately adjacent to each other, short-read-based methods would have difficulty assigning the reads near gene boundaries, while Nanopore long reads from adjacent genes can be easily separated (Fig. 1e).

Genome-wide analysis showed that the poly(A) transcripts are the most abundant forms of the four, followed by readthrough transcripts and 5′ cleavage products (Fig. 1c, d). The 3′ cleavage products only occupied a low proportion (1%) among the RNA intermediates, consistent with the rapid degradation of uncapped transcripts by exonuclease AtXRN3 in Arabidopsis [14, 47, 48]. FLEP-seq monitors both co-transcriptional cleavage and readthrough, whereas other methods do not. Therefore, by analyzing the 3′ end of the non-poly(A) RNAs, our results showed a clear pattern for termination: with a sharp peak enriched at the poly(A) site followed by a gradual decline (Fig. 1f). Taken together, the full-length nascent RNA sequencing method is well suited for tracking various transient RNA products during transcription termination.

### The termination landscape of Arabidopsis genes

**Fig. 2:**
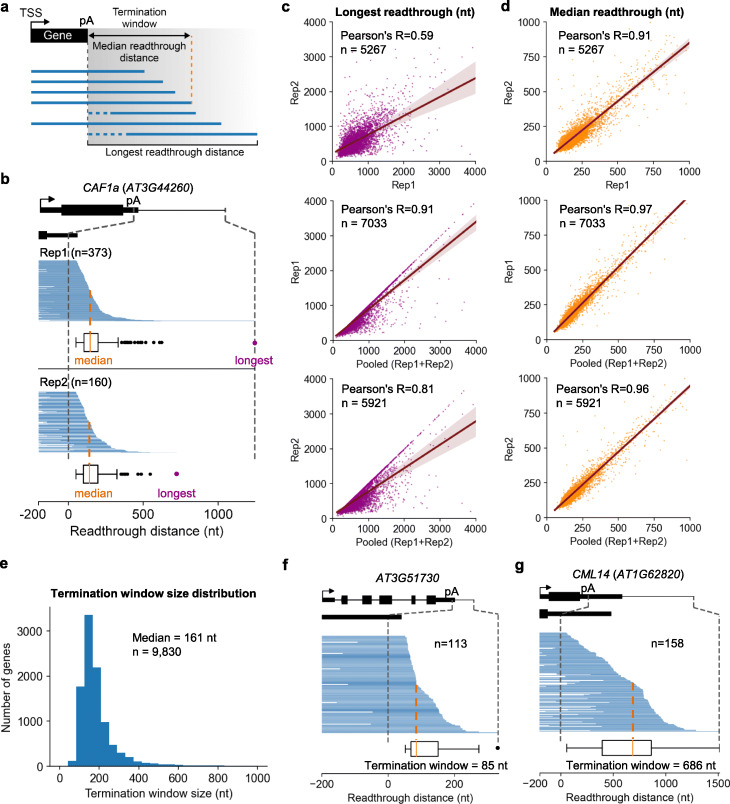
The termination landscape in Arabidopsis. **a** Schematic diagram shows the definition of termination window presented in this study. Reads with 3′ end located more than 50 nt downstream of poly(A) site are used to define termination window. The readthrough distance is calculated as the distance between the poly(A) site and the 3′ end of reads. The median readthrough distance at the corresponding gene is used to represent its termination window size. **b** Comparison of termination window size quantified by either the median or longest readthrough distance. The gene model is shown at the top and is zoom-in to highlight the region from 200 nt upstream of the poly(A) site to the longest readthrough distance. The boxplots show the readthrough distance distribution for all reads in the region. pA, poly(A) site (also in Fig. 2f, g, and Fig. 4a, d). **c, d** Scatter plots for the longest (**c**) and median (**d**) readthrough distance per gene between replicates (a minimum of 15 readthrough reads for calculating the termination window size). The Pearson’s correlation coefficient and the number of genes (*n*) are shown. **e** The distribution of termination window size among genes. **f, g** Examples of genes with short and long termination windows, respectively

Transcription termination of Pol II is thought to be a random process that occurs at different distances downstream of the poly(A) site [18, 49]. We refer to the distance in which Pol II traveled past the poly(A) site before released from DNA as the previously described “termination window (TW)” [15, 17], also known as “termination zone” [18, 50]. To date, the genome-wide measurement of termination window has only been accessed by short-read sequencing, including in human cells with TT-seq method [17], in yeast with 4tU-seq method [15], and in Arabidopsis with plaNET-seq method [42]. The TT-seq study found a wide range of termination windows among ~ 7000 genes in human cells, with a median width of ~ 3300 bp and can go up to over 10 kb [17]. The 4tU-seq study found that the median termination window is 163 bp in yeast [15]. The plaNET-seq study suggested a median readthrough distance of ~ 500 bp for Arabidopsis genes [42]. Compared to these methods, our FLEP-seq method can capture and distinguish the various forms of full-length terminating RNAs with unprecedented depth and resolution, thus presents an opportunity to examine termination patterns for individual genes in detail. For each nascent RNA molecule, readthrough can be calculated as the distance between its 3′ end of the RNA and the poly(A) site of the gene (Fig. 2a). Because the detection of the longest readthrough RNA at any loci will be correlated with sequencing depth, using the longest readthrough distance to represent the termination window will make the estimation sensitive to fluctuations of sequencing depth (Fig. 1d, Fig. 2b, c). Our single-molecule long-read method can track each RNA molecule separately, therefore enabling us to use the median readthrough distance of each gene to represent its termination window size as a more robust measurement (Fig. 2b, d). We observed consistent TW size (Pearson’s *R* = 0.91) for individual genes between the two biological replicates of FLEP-seq libraries (Fig. 2d). Hence, we use the median readthrough distance to represent the TW size in the following analyses. We found the lengths of TWs vary drastically among the 9830 Arabidopsis genes analyzed (each with a minimal of 15 readthrough reads), ranging from ~ 50 nt to over 1000 nt (Fig. 2e), with the median size at ~ 160 nt (Additional file 2, Table S1). For example, the termination of gene *AT3G51730* occurred quickly downstream of the poly(A) site with a TW size of 85 nt (Fig. 2f), while gene *AT1G62820* (*CML14*) has many longer readthrough transcripts with a TW size of 686 nt (Fig. 2g). Even using the longest readthrough to represent TW, the median of which is 521 nt, the TW in Arabidopsis is still considerably shorter than the TW in human reported by TT-seq [17]. The much shorter termination windows of Arabidopsis compared to human may be due to the compact genome size of Arabidopsis, which is ~ 20 times smaller, and hence has a much denser gene arrangement. Taken together, our data reveals a comprehensive termination landscape of Arabidopsis.

### Pol II co-localizes with termination factors at the end of termination window

**Fig. 3:**
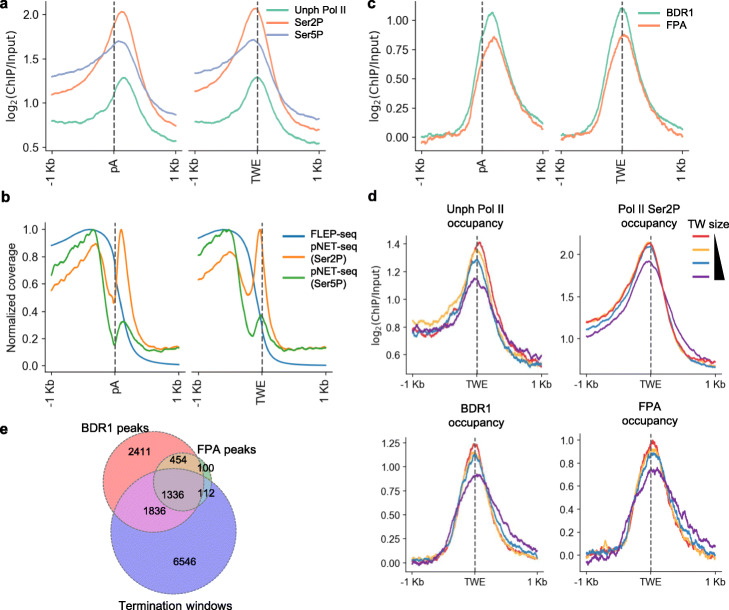
The end of termination window precisely matches Pol II occupancy and co-localizes with termination factors. **a, c** Meta-profile of published ChIP-seq signal for Pol II, Ser2P, Ser5P [53] (**a**) and BDR1, FPA [54] (**b**) centered around either the poly(A) site (pA) or the termination window end (TWE). **b** Meta-profile of read coverage for FLEP-seq and pNET-seq [41] centered around the poly(A) site or the termination window end. **d** Meta-profiles of Pol II, Pol II Ser2P, BDR1, and FPA ChIP-seq signal in four groups of genes ordered by different termination window sizes. **e** Venn diagram shows the overlap between BDR1, FPA peaks, and termination windows

The transition from transcription elongation to termination requires the slow down or pause of the elongating Pol II [51]. The pausing of Pol II at the 3′ end of the gene is usually associated with Pol II carboxy-terminal domain (CTD) serine 2 phosphorylation (Ser2P) [2, 41]. Previous studies revealed that the Pol II accumulation downstream of poly(A) site is related to the 3′ pausing [18, 50, 52]. Compared to previously published ChIP-seq data [53], we observed that ends of the termination window precisely reside with the peaks of Pol II and Ser2P, further suggesting that using median instead of the longest readthrough distance to represent TW is a more appropriate measurement (Fig. 3a). The presence of termination window engaged Pol II peak could also be observed in pNET-seq [41] and GRO-seq [40] data (Fig. 3b; Additional file 1, Fig. S3a).

During the termination process, many factors are associated with the Pol II elongation complex [1]. We found that BDR1, a negative elongation factor that prevents transcriptional interference in Arabidopsis [54], localized precisely at the end of the termination window (Fig. 3c). Similarly, an mRNA 3′-end processing factor FPA that controls the cleavage and polyadenylation [12, 55, 56] is found to coincide with BDR1 distribution (Fig. 3c). In addition, ATAC-seq data [57] showed a preference for open chromatin status at the end of termination window (Additional file 1, Fig. S3b), consistent with the previous report that nucleosome depletion is linked to the 3′-end formation [58]. While poly(A) sites are featured with low CG methylation and low GC content compared to flanking regions, we did not observe obvious change of DNA methylation or GC contents at major site for transcription termination (TWE) (Additional file 1, Fig. S3c, d). We also found that the strength of Pol II occupancy is negatively correlated with TW size (Fig. 3d), and similar trends were observed in the distribution of BDR1 and FPA. Previous study demonstrated that BDR proteins prefer to reside at gene borders, in both transcription start sites (TSS) and transcription end site (TES) [54]. It is worth to clarify that poly(A) site, TES, and TWE are different from each other—poly(A) site is where cleavage and polyadenylation occurs; TES in Araport11 is defined based on the reconstructed transcript assembly using a collection of Illumina RNA-seq [19], and TWE is the median readthrough length defined by FLEP-seq data (Additional file 1, Fig. S1d). Our results further revealed that most of FPA peaks overlapped with BDR1 peaks at termination window (Fig. 3e). Nevertheless, only a small portion of termination windows contained BDR1 and FPA peaks at their ends (Fig. 3e), suggesting many other factors are potentially involved in the termination process in Arabidopsis.

### tRNA genes promote efficient termination of Pol II transcription

**Fig. 4:**
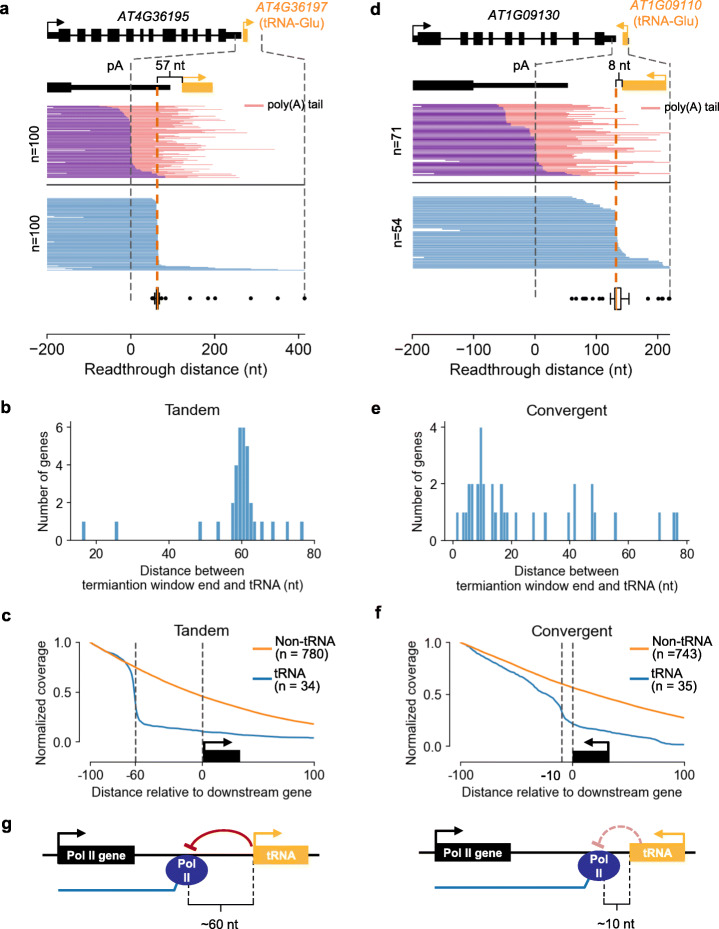
Downstream tRNA promotes efficient termination of upstream protein-coding genes. **a, d** Examples of Pol II genes that have tRNA gene immediately downstream, arranged in tandem (**a**) or convergent (**d**). The number of reads (*n*) is shown. The poly(A) transcript reads are shown in purple, and their poly(A) tail is shown in red. The readthrough transcript reads are shown in blue. The distance between the termination window end and tRNA is marked by bracket. **b, e** The distribution for the distance between each pair of the termination window end (TWE) and its downstream tRNA gene. Tandem orientation (**b**). Convergent orientation (**e**). **c, f** Read coverage of genes that have tRNA or non-tRNA genes immediately downstream. In the case of tandem orientation, the start of the downstream gene is used as a reference (**c**). In the case of convergent orientation, the end of the downstream gene is used as a reference (**f**). **g** Model for tRNA-dependent termination in Arabidopsis

The tRNA genes are transcribed by Pol III [59]. In this study, we discovered a termination mechanism for Pol II that is shaped on the downstream tRNA genes. In the compact Arabidopsis genome, we identified in total more than 60 Pol II genes that have adjacent tRNA genes immediately downstream (distance to downstream gene < 200 nt) (Additional file 3, Table S2). In the tandemly arrange case, the readthrough transcripts terminated sharply at 60 nt upstream of the tRNA gene, and the distance between termination window end and downstream tRNA is consistently at ~ 60 nt (Fig. 4a, b). Our results showed that this highly efficient pattern of termination is specific to genes to which a tRNA gene locates downstream (Fig. 4c; Additional file 1, Fig S4). For the 34 genes whose termination is regulated by the downstream tRNA in tandem direction, 26 of them have termination window ends located ~ 60 nt upstream of the tRNAs. While in the convergently arrange cases, the distance between the termination window end and tRNA is around 10 nt with a less obvious concentration (Fig. 4d, e), and these genes also terminated more efficiently than those with non-tRNA immediately downstream (Fig. 4f). Previous report estimated that RNA polymerase may contact up to 90 bp of DNA (− 70 to + 20) at promoter regions [60]. Moreover, previous literature has shown that Pol II pausing or stalling can be influenced by chromatin structure [2]. Therefore, it is possible that the termination of Pol II elongation at − 60 nt is caused by a unique chromatin status at the tRNA gene promoter. To test this scenario, we analyzed published data of MNase-seq [61], ChIP-seq (H3K4me3, H3K36me3, H3K27me3, H3K9me2) [62], and the CG, CHG, and CHH DNA methylation level to check the chromatin status at the two tRNA loci showed in Fig. 4, including nucleosome positioning, histone modification, and DNA methylation (Additional file 1, Fig. S5). We did not observe an obvious enrichment or depletion of the common epigenetic marks around the tRNA promoters. Future studies on the factors that bind to the promoter of tRNA gene may help to explain its role in blocking Pol II transcription. Besides Arabidopsis, a previous report in *Leishmania major* also found tRNA gene can serve as terminator for Pol II transcription in the trypanosomatida [63].

We also analyzed the correlation between termination window size and intergenic distance, direction, and transcription level of downstream protein-coding and/or noncoding genes, respectively. The genome-wide analysis showed that the termination window size of genes arranged in tandem is similar to genes arranged in convergent direction (Additional file 1, Fig. S6a). We found that termination window size is slightly positively correlated with intergenic length and slightly negatively correlated with the transcription level of downstream genes (Additional file 1, Fig. S6b-d). We proposed that the pairing between Pol II gene and their downstream tRNA may be evolutionarily beneficial for assisting efficient termination in a small and compact genome (Fig. 4 g).

### Mutation of *AtXRN3* delays the termination of cleaved readthrough transcription

Many previous studies have supported the torpedo model for transcription termination, in which a nuclear 5′ → 3′ exonuclease plays the central role in degrading the 3′ cleavage products, and eventually catch up with elongating Pol II to expel it from DNA template via kinetic competition [2, 16, 18]. In Arabidopsis, two nuclear exonucleases AtXRN2 and AtXRN3 are orthologs of the human Xrn2 [11, 48]. Although they share extensive sequence similarities, only AtXRN3 is shown to be the primary exonuclease involved in Pol II termination [14, 47, 64]. RNA-seq analysis performed with total RNAs from weak alleles of *atxrn3* show increased signal of mostly polyA+ mRNAs downstream of poly(A) site compared to wildtype [14, 47]. However, it remains unclear if the 3′ cleavage products without poly(A) tails accumulated in *atxrn3* and how *atxrn3* affects readthrough, due to the limitation of short-read sequencing. We set out to address these questions by taking advantage of our full-length nascent RNA sequencing method, which can capture the 3′ cleavage products, particularly those without poly(A) tails, enriched in the partial loss-of-function allele of *atxrn3*. In order to maximize the capture rate of 3′ cleavage products which might not be closely associated with chromatin, we used nuclear RNA instead of chromatin-bound RNA as the nascent RNA input of FLEP-seq. It turned out that both the nuclear fraction and the chromatin-bound fraction can efficiently capture the cleaved and readthrough transcripts (Additional file 1, Fig. S7a). Since nuclear RNA isolation involves fewer steps than chromatin-bound RNA isolation, we proceeded with nuclear RNA to make FLEP-seq libraries in a series of mutants and wildtype controls (please see “Methods” for detail). The termination window sizes from wildtypes using chromatin-bound RNA and nuclear RNA are highly consistent (Additional file 1, Fig. S7b), suggesting that nuclear RNA is a viable substitute for chromatin-bound RNA in studying transcription termination in Arabidopsis.

**Fig. 5:**
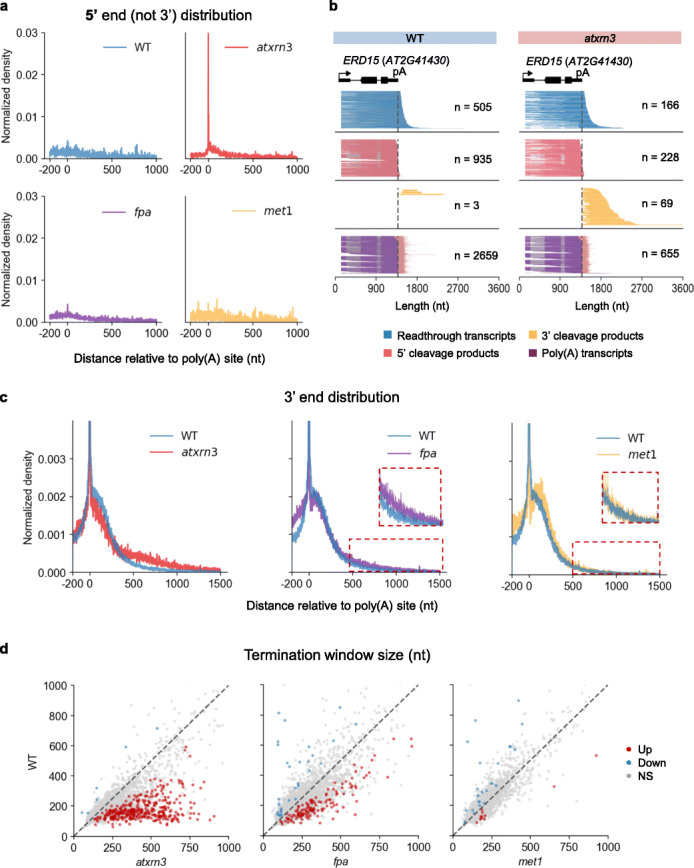
3′ cleavage products accumulate in the *atxrn3* mutant. **a** 5′ end distribution for the non-poly(A) reads in wildtype *atxrn3*, *fpa*, and *met1* mutant near poly(A) site. **b** Examples of genes with accumulated 3′ cleavage products in *atxrn3* mutant. Left panel, wildtype; right panel, *atxrn3* mutant. **c** Comparison of 3′ end distribution for the non-poly(A) reads in wildtype and *atxrn3*, *fpa*, *met1* mutant near poly(A) site (the maximum limit of the y-axes is set at 0.003 to highlight differences at low-signal region). The zoomed-in view is shown in red dashed box. **d** Comparison of termination window size per gene between wildtype and mutants. The *p* value was calculated using a Mann–Whitney *U* test. NS, not significant (*p* > 0.001)

Compared to wildtype, the 3′ cleavage products drastically accumulated in the *atxrn3* mutant (Fig. 5b), consistent with the function of AtXRN3 in 5′ → 3′ degradation of co-transcriptional cleavage products [9, 11, 14]. This accumulation leads to a clear peak of the 5′ end of cleaved readthrough reads (3′ cleavage products) at poly(A) site in *atxrn3* mutant, which is absent in the wildtype control library (Fig. 5a). Similar results were also observed in human Xrn2 depletion cell line detected by short-read-based method POINT-5 [25]. In addition, accumulation of the 3′ cleavage products is not influenced by splicing, as genes with or without splicing have 3′ cleavage products enriched in *atxrn3* and aligned accurately at the poly(A) site (Fig. 5b, Fig. S8). From a genome-wide perspective, we compared the size of termination windows in the *atxrn3* mutant and in wildtype, and the result showed a strong impact of *atxrn3* on termination window size at hundreds of gene loci (Fig. 5d). The termination window of 354 genes were statistically longer in the *atxrn3* mutant than in wildtype (Mann–Whitney *U* test, *p* value < 0.001). These results illustrated that AtXRN3 is specifically responsible for the degradation of 3′ cleavage products in vivo.

In addition to *atxrn3*, we also characterized the *fpa* and *met1* mutants by FLEP-seq. FPA is a component of the 3′ end processing complex, and MET1 is the key DNA methyltransferase in Arabidopsis [65]. The *fpa* mutant FLEP-seq data showed a global impact on termination window size and a prolonged 3′ end distribution compared to WT (Fig. 5c, d), consistent with its previously reported function as a termination factor [12, 56]. We also found some interesting cases that support the previous report of chimeric transcripts and cryptic splicing occurring in the *fpa* mutant [12, 56]. For example, some readthrough transcripts in the *fpa* mutant can extend into downstream genes to form chimeric RNAs accompanied by the cryptic splicing event (Additional file 1, Fig. S9a). Cryptic splicing event in the *fpa* mutant can even result in excision of the entire gene in the middle from the readthrough transcript that spans multiple genes (Additional file 1, Fig. S9b). In the *met1-1* mutant of DNA methyltransferase MET1 [65], in which most CG methylation at genic regions is lost (Additional file 1, Fig. S13a), the distribution for 3′ ends of readthrough RNAs is largely unaffected (Fig. 5c), and the size of termination windows is similar to that in wildtype Col-0 (Fig. 5d). However, it is worth noting that *met1-1* is not a null allele and still have some remaining CG methylation at TE regions (Additional file 1, Fig. S13b), recent characterization using ONT direct RNA sequencing of full-length mRNA from the strong *met1-3* allele, which removes virtually all CG methylation, has discovered the effects of DNA methylation on splicing site and poly(A) site selection, as well as on poly(A) tail length [66].

**Fig. 6:**
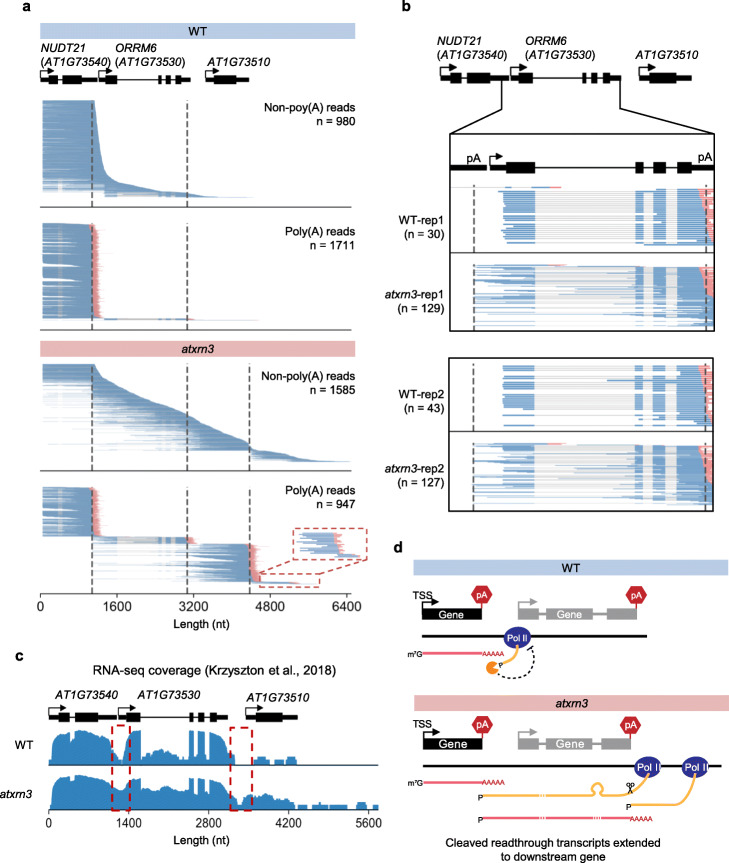
Cleaved readthrough transcripts can transcribe into downstream genes and produce spliced and polyadenylated mRNAs in *atxrn3*. **a** Examples of genes with cleaved readthrough reads extended to downstream genes. Upper panel, wildtype; lower panel, *atxrn3* mutant. The black dashed line indicates the poly(A) site. The red dashed box showed the magnified view of cleaved and polyadenylated reads at the subsequent poly(A) site. **b** Zoomed-in view of poly(A) reads aligned to gene *AT1G73530* in two biological replicates of the wildtype and *atxrn3* FLEP-seq libraries. **c** Read coverage of RNA-seq data [14] around the *AT1G73540-AT1G73510* region in wildtype and *atxrn3* mutant (log_2_ scale). **d** Model of AtXRN3-mediated transcription termination

Furthermore, we identified 14 genes with cleaved readthrough transcripts entering their immediate downstream genes in *atxrn3* mutant (Additional file 4, Table S3). For example, *AT1G73510* is a pollen-specific gene that is not expressed in seedlings (Additional file 1, Fig. S10), the materials used in our FLEP-seq libraries. In *atxrn3*, readthrough from its upstream gene *NUDT21* (*AT1G73540*) and *ORRM6* (*AT1G73530*) can continue elongation and pass through the entire downstream gene, and then be cleaved and polyadenylated (Fig. 6a). Strikingly, our full-length data revealed that the readthroughs in *arxtn3* can be cleaved and polyadenylated multiple times as they elongate through several subsequent poly(A) sites in a row (Fig. 6a, magnified view). In addition, we found that cleaved readthrough transcription can yield normally spliced and polyadenylated mRNAs without their own transcription initiation (Fig. 6a). This can be clearly seen with a zoomed-in view around the TSS site of *AT1G73530* in two biological replicates of the wildtype and *atxrn3* FLEP-seq libraries, showing that some polyadenylated transcripts of *AT1G73530* in *atxrn3* originated from the upstream poly(A) site as cleavage products of upstream transcriptional readthrough, instead of their own initiation (Fig. 6b). A reordered view of reads at *AT1G76180*-*AT1G76170* region also confirms this observation, with most reads of the downstream *AT1G76170* come from the cleaved readthrough of the upstream gene *AT1G76180* (Additional file 1, Fig. S11). Besides nascent RNA, we also check the mRNA level by analyzing previously published RNA-seq data of wide-type and *atxrn3* mutant [14]. The coverage plot confirmed that more reads are aligned to the intergenic region of *AT1G73540*-*AT1G73530* in *at*xrn3 mutant, compared to the fewer reads at the same regions in wildtype (Fig. 6c). It remains unclear if these mRNAs originated from 3′ cleavage products can be translated, as they may lack the 5′ cap structure. Previous work on *AtXRN3* proposed several models to explain the elevated poly(A) + RNA-seq signal downstream of poly(A) site, including a role for transcription activation of downstream genes by readthrough transcription [14, 47]. Our single-molecule nascent RNA data suggests that readthrough transcription itself could be enough to drive the production of multiple downstream transcripts, highlighting the importance of AtXRN3-mediated transcription termination in the compact Arabidopsis genome (Fig. 6d).

However, the *AT1G73530*-*AT1G73510* fusion poly(A) transcripts cannot be solely explained by the loss of 5′ to 3′ exonuclease activity as AtXRN3 should not affect the cleavage at the poly(A) site. It is possible that AtXRN3 may affect chromatin status that in turn determines poly(A) site selection and the readthrough phenomena. To check if DNA methylation status is altered at poly(A) sites in the *atxrn3* mutant, we performed whole-genome bisulfite sequencing (WGBS) in seedlings of *atxrn3* and corresponding Col-0 control. At the *AT1G73530*-*AT1G73510* loci, there is little CG methylation in the wildtype, and it remains mostly unmethylated in the *atxrn3* mutant (Additional file 1, Fig. S12a). This is consistent with previous DNA methylation profiling of multiple wildtype Arabidopsis libraries from the Jacobsen group [67] (Additional file 1, Fig. S12b). In addition, we found that the overall DNA methylation pattern at genic regions remains unchanged in the *atxrn3* mutant compared to WT in CG, CHG, and CHH contexts (Additional file 1, Fig. S13c). However, individual poly(A) sites could still be affected, as a recent work has discovered large number of novel TTSs in the *met1-3* mutant, suggesting a role of DNA methylation in poly(A) site selection [66]. We proposed that, besides exonuclease activity of AtXRN3, other factors such as chromatin status and DNA modification could also contribute to the accumulated readthrough transcripts in the *atxrn3* mutant.

## Discussion

Here we applied single-molecule Nascent RNA sequencing to investigate the transcription termination landscape at a genome-wide level in Arabidopsis. The single-molecule feature of Nanopore sequencing enables our FLEP-seq to analyze the termination window of genes (Fig. 2e), regardless of how narrow the intergenic regions are (Fig. 1e). In addition, our data allows the precise and robust identification of poly(A) sites in the same FLEP-seq library (Fig. 1b; Additional file 1, Fig. S1), which can facilitate the accurate analysis of PAS-dependent termination. Besides, in stress or mutant conditions, 3′-end cleavage may occur at novel poly(A) sites [43]. Therefore, FLEP-seq is suitable to study PAS-dependent termination under different conditions and genetic backgrounds.

Many powerful Illumina-based methods have been applied to characterizing transcription termination, including GRO-seq, NET-seq, 4tU-seq, and TT-seq [15, 17, 21, 24]. Several protocols also take advantages of the paired-end sequencing function of Illumina to distinguish the 5′ and 3′ ends of each RNA molecule, such as TIF-seq [68]. The Illumina platform also holds the advantage of lower cost therefore easier to achieve deeper sequencing yields. Nevertheless, our Nanopore-based single-molecule approach has several unique advantages compared to previous Illumina-based methods—(a) There is no RNA fragmentation step involved in the FLEP-seq procedure; therefore, it can capture the full-length RNA intermediates during termination, with information on both their 5′ end and 3′ end positions, as well as the poly(A) tail; (b) FLEP-seq can separate the cleaved and uncleaved readthrough RNAs that are previously indistinguishable in the short-read sequencing datasets; (c) The single-molecule measurement enables us to quantify the termination window using median readthrough distance, which is less vulnerable to fluctuations in sequencing depth compared to coverage-based assessment from previous Illumina-based methods. Hence, with these unique features, FLEP-seq could serve as a new method for analyzing transcription termination that is complementary to previous Illumina-based methods. Besides, metabolic-labeling-based methods, such as 4tU-seq and 4sU-seq, can detect newly synthesized RNA and can be combined with long-read sequencing platforms to characterize full-length nascent RNA [34].

Pol II pausing after poly(A) site is a common feature in plant transcription [39, 41]. Previous studies have proposed that Pol II pausing downstream of poly(A) site promotes the mRNA 3′-end processing and subsequent torpedo degradation for termination [18, 69]. Consistent with this idea, we observed that the 3′-end processing factor FPA and negative elongation factor BDR1 co-localize with Pol II at a set of genes (Fig. 3). Future application of FLEP-seq to study termination changes in the mutants of the termination factors, such as BDR1 and CPSF components, could further expand our understanding of the termination mechanism in plants. Another important question is what are the potential determinants that make hundreds of genes sensitive to *atxrn3* mutation. We do not have a clear answer yet, but it is worth noting that the *atxrn3* alleles we and other previous studies sequenced are weak alleles, and the strong allele of *atxrn3* is embryo lethal [48], which suggests AtXRN3 may have a much stronger impact than revealed from the weak alleles.

FLEP-seq approach has its limitations [38]. For example, when RNA is reverse transcribed into cDNA, the integrity of long reads relies on the success of a complete reverse transcription, which favors shorter RNAs. This problem is particularly relevant for animal nascent RNAs with long introns, and recent advancements in Nanopore direct RNA sequencing technology may help to improve this shortcoming [26, 30, 33, 34, 56]. However, the current generation of ONT DRS technology has several issues that limit its use in characterizing transcription termination: (a) The current ONT DRS kit is designed for profiling polyA+ RNAs, additional steps such as in vitro polyadenylation [33, 34], or using customer-specific RT adaptor [70, 71], can be applied to characterize non-polyA RNAs; (b) DRS has much lower yields per run (~ 1 million reads per flow cell on MinION) compared to cDNA sequencing (~ 10 million reads per flow cell on MinION); (c) DRS requires much higher input (500 to 1000 ng) compared to the as little as 1 ng needed for FLEP-seq. We hope that the future generation of ONT DRS will be able to address these issues so that it can be more easily applied on non-polyA RNAs.

## Conclusions

Our single-molecule full-length nascent RNA sequencing approach presented here has the unique advantage of distinguishing the full-length readthrough, cleaved, and polyadenylated forms of transient RNA intermediates in the termination process and enables the characterization of transcription termination patterns at the single-gene level in the model plant Arabidopsis.

## Methods

### Plant materials and growth conditions

*Arabidopsis thaliana* wildtype (accession Columbia-0) and T-DNA insertion line of *atxrn3* (SALK_116909C), *fpa* (SALK_011615), and *met1-1* [65] were used in this study. Seeds were grown on 1/2 MS medium at 22 °C (16 h light–8 h dark) for 12 days before collection. Twelve-day-old seedlings were harvested and immediately frozen in liquid nitrogen, then stored at − 80 °C for RNA extraction.

### Nuclear RNA extraction and FLEP-seq library construction

Nuclear RNA extraction is performed according to the previously reported method [37, 38]. In brief, 2 g of seedlings was ground with liquid nitrogen and transferred to an ice-cold RNase/DNase-free 50-ml tube with 10 ml Honda buffer (0.44 M sucrose, 1.25% (w/v) Ficoll, 2.5% (w/v) dextran T40, 20 mM HEPES-KOH pH 7.4, 10 mM MgCl_2_, 0.5% (w/v) Triton X-100, 1 mM dithiothreitol (DTT), 1× protease inhibitor (Roche), and 100 ng μl^−1^ tRNA). The samples were homogenized and filtered through Miracloth, and the remaining samples on filter were washed with another 10 ml Honda buffer. After centrifuged at 2000*g* for 5 min at 4 °C, the nuclear pellets were resuspended and washed twice with 15 ml Honda buffer. The pellets were transferred to a 1.5-ml RNase/DNase-free microcentrifuge tube in 1 ml Honda buffer and centrifuged at 8000*g* for 1 min at 4 °C. Then the supernatant was completely removed. For RNA extraction, the nuclei pellet was resuspended with 1 ml TRIzol and vortexed to mix thoroughly. After 10 min incubation at room temperature, 0.2× volume of chloroform was added, mixed, and incubated at room temperature for 5 min. The mixture was centrifuged at 14,000*g* for 10 min at 4 °C. The supernatant was collected and added with one volume of 100% (w/v) ethanol. The RNA extraction was performed according to the manufacturer’s instructions (ZYMO, R2070). RNA concentration was measured by Nanodrop and Qubit 3.0 fluorometer with Equalbit RNA HS assay kit (Vazyme, EQ211-01).

At least 2 μg nuclear RNA was used for FLEP-seq library preparation. Ribosomal RNA (rRNA) was depleted using riboPOOL kit (siTOOLs Biotech) following the manufacturer’s instructions. After purification with the ZYMO RNA Clean & Concentrator-5 kit (ZYMO, R1013), the nuclear RNA was mixed with 50 pmol 3′ adapter (5′-rAppCTGTAGGCACCATCAAT–NH2-3′, NEB, S1315S) at 65 °C for 5 min, and then placed on ice for more than 1 min. Then, 2 μl 10× T4 RNA ligase reaction buffer (NEB, M0242), 10 μl 50% PEG 8000 (NEB, M0242), 1 μl 40 U μl^−1^ Murine Rnase Inhibitor (Vazyme, R301-03), and 1 μl T4 RNA ligase 2, truncated K227Q (NEB, M0242) were added to the RNA and mixed thoroughly. The ligation reaction was performed at 16 °C for 10 h. After that, the RNA was purified and concentrated to 6 μl using the ZYMO RNA Clean & Concentrator kit (ZYMO, R1013). Reverse transcription and template-switching were performed using the SMARTer PCR cDNA Synthesis Kit (Takara, 634926) with minor modification. The SMART CDS Primer II A was replaced by the custom 3′ cDNA RT primer (5′-AAGCAGTGGTATCAACGCAGAGTACATTGATGGTGCCTACAG-3′), which is complementary to the 3′ adapter sequence. To minimize the PCR bias resulting from over-amplification, PCR cycle optimization was performed to determine the best cycle number for cDNA amplification. The cDNA for Nanopore library construction was amplified using the optimized cycle number and purified twice with the VAHTS DNA Clean Beads (Vazyme, N411). DNA concentration and quality were measured by Qubit 3.0 and Agilent 2100 Bioanalyzer. Then, 200 fmol DNA was used to construct the Nanopore library using the Ligation Sequencing Kit (SQK-LSK109, Oxford Nanopore Technologies) according to the instruction. Libraries were loaded onto R9.4 flow cell (Oxford Nanopore Technologies) and sequenced on MinION device for ~ 48 h.

### Nanopore data processing

The Nanopore data analysis workflow is provided in Additional file 1, Fig. S2. The Nanopore data pre-processing was performed as previously described with minor modification [37, 38]. Guppy basecaller (Oxford Nanopore Technologies, v4.0.11) was used to convert Nanopore raw signal to sequence, with parameters *--c dna_r9.4.1_450bps_hac.cfg*, *--qscore_filtering*. The basecalled reads were mapped to Arabidopsis TAIR10 genome using Minimap2 v2.10-r761 [72] with parameters: *-ax splice*, *–secondary = no*, *-G 12000*. The unmapped reads, not primary alignment reads, or supplementary alignment reads were removed using SAMtools [73] view with parameter *-F 2308*. The 5′ or 3′ unmapped sequences (soft-clip sequences) plus the flanking 20 nt mapped sequences were used to search the template-switching oligo sequence (5′-AAGCAGTGGTATCAACGCAGAGTACATGGG-3′) and the 3′ adapter sequence (5′-ATTGATGGTGCCTACAG-3′) using our custom script adapterFinder.py. The template-switching oligo and the 3′ adapter can characterize the integrity of 5′ and 3′ end of reads and provide the strand information. Therefore, only reads containing both sequences were used for subsequent analysis. PolyAcaller was used to estimate poly(A) tail lengths of Nanopore reads [38].

### Identification of poly(A) sites

The identification of poly(A) sites was performed as previously described [45]. Briefly, the 3′ end of poly(A) transcript read was considered as the poly(A) site. Due to the heterogeneity of poly(A) sites within the same gene in Arabidopsis, the poly(A) sites within 24 nt of each other within the same gene were grouped into a poly(A) site cluster (PAC). The PAC with less than three poly(A) sites was discarded. The poly(A) site with the greatest number in the PAC was defined as the representative poly(A) site. The representative poly(A) sites were used in the subsequent analysis. To eliminate the influence of alternative polyadenylation, only genes with a single representative poly(A) site were used for further analysis.

### Classification of RNA intermediates produced during termination

The schematic of RNA intermediates is shown in Fig. 1a. In detail, reads are categorized as “poly(A) transcripts” if their poly(A) tail lengths are greater than 15 nt as previously described [37, 38]. To eliminate the influence of sequencing error on the 3′ end accuracy, the remaining reads are considered as non-poly(A) reads if the distance between the 3′ adapter and the mapping region is less than 5 nt. Pysam [73] (https://github.com/pysam-developers/pysam) was used to extract the 5′ and 3′ end coordinates of reads from BAM files. Non-poly(A) reads with 5′ ends located at gene body ( > 50 nt upstream of poly(A) site) and 3′ end located more than 50 nt downstream of the poly(A) site are considered as “readthrough transcripts.” Non-poly(A) reads with 5′ ends located at gene body as well as 3′ ends within 50 nt upstream and downstream of poly(A) sites are considered as “5′ cleavage products.” Non-poly(A) reads with 5′ end within 500 nt downstream of poly(A) sites are regarded as “3′ cleavage products.” Of note, when two neighboring genes are arranged closely on the same strand, the “3′ cleavage products” is considered only if the 5′ end is 100 nt before the TSS of the downstream gene.

### Calculation of termination window size

The readthrough distance was defined as the distance between poly(A) site and 3′ end of each read (Fig. 2a). Genes with a minimum read count threshold of 15 (readthrough transcript reads + 3′ cleavage product reads) were used. For each gene of interest, we use the median readthrough distance to represent the termination window size, to reduce the effect of sequencing depth (Fig. 2b–d).

### Whole-genome bisulfite sequencing and analysis

Genomic DNA was extracted from 12-day-old seedlings of wildtype and *xrn3* mutant using Hi-DNAsecure Plant Kit (TIANGEN, Cat. DP350-02). The whole-genome bisulfite sequencing (WGBS) libraries were prepared and sequenced at the BGI Group on the MGISEQ-2000RS. For each library, adapters were trimmed by using fastp v0.20 [74], and reads were mapped to Arabidopsis TAIR10 genome using BSMAP [75]. The DNA methylation level in each 100 bp bin was calculated as the ratio of methylated cytosines to the total number of cytosines (#C/(#C + #T)) [67].

### Illumina data processing

Illumina reads were first trimmed the adapter sequences using fastp v0.20 [74]. For the pNET-seq, plaNET-seq, and GRO-seq data, the reads were mapped to the Arabidopsis TAIR10 genome using STAR v2.7.2b [76]. Only the uniquely mapped reads with mapping quality greater than 10 were used for further analysis. For ChIP-seq and ATAC-seq data, reads were mapped to Arabidopsis TAIR10 genome using Bowtie2 v2.3.5.1 [77], with parameters *-dovetail*, and with parameters *-X 1000* for paired-end sequencing data to set the maximum insert size. The duplicate reads were removed by using Picard v2.2.4 in MarkDuplicates mode (http://broadinstitute.github.io/picard/). For ChIP-seq data, input-normalized log_2_ fold enrichment was computed using deepTools bamCompare [78], with parameters *--binSize 10 --scaleFactorsMethod SES*. ChIP-seq peaks were called on input and control BAM files with MAC2 [79]. Normalized coverage was computed using deepTools bamCoverage [78], with parameters *--binSize 10 --normalizeUsing RPGC --effectiveGenomeSize 119481543*. The normalized coverage was directly used to produce meta-profile without smoothing.

### Quantification and statistical analysis

Pearson’s correlation coefficient is used to measures the statistical relationship. Mann–Whitney *U* test was used to obtain the significance between conditions. All information about statistical testing can be found in figure legends. This includes the number of observations, statistical tests used, and significance level.

## Supplementary Information

**Additional file 1: Figures S1-13.** Supplementary figures

**Additional file 2: Table S1.**The termination window size and poly(A) site identified by FLEP-seq data.

**Additional file 3: Table S2.** Closely spaced genes with tRNA located immediately downstream.

**Additional file 4: Table S3.** Genes with cleaved readthrough transcripts enter the downstream genes in *atxrn3*.

**Additional file 5.** Review history.

## Data Availability

The nuclear RNA FLEP-seq and WGBS data generated in this paper have been deposited in the Genome Sequence Archive in National Genomics Data Center, China National Center for Bioinformation / Beijing Institute of Genomics, Chinese Academy of Sciences, under accession number CRA005016 and CRA005017 [80]. The chromatin-bound RNA FLEP-seq data have been deposited in NCBI’s Sequence Read Archive (SRA): PRJNA591665 [37, 38]. The FLEP-seq data are also displayed in a JBrowse (https://zhailab-sustech.github.io/jbrowse2/). All other relevant data supporting the key findings of this study are listed as follows. GRO-seq data were acquired from published studies (GEO accession: GSE83108, GSE100010, and GSE109974) [39–41]. pNET-seq and plaNET-seq data were obtained from published studies (GEO accession: GSE109974 and GSE131733) [41, 42]. Pol II, Ser2P, FPA, and BDR1 ChIP-seq data were obtained from published studies (GEO accession: GSE95301 and GSE112443) [53, 54]. ATAC-seq data were obtained from the published studies (GEO accession: GSE85203) [57]. The pipeline developed for pre-processing FLEP-seq data is available at https://github.com/ZhaiLab-SUSTech/FLEPSeq. All scripts used for transcription termination analysis in this study are available at Github (https://github.com/ZhaiLab-SUSTech/Termination_landscape) [81] or Zenodo (10.5281/zenodo.5607518) [82].
