# Supplementary material for: Landscape of transcription termination in Arabidopsis revealed by single-molecule nascent RNA sequencing
Source: Genome Biol. 2021 Nov 25;22:322. doi: 10.1186/s13059-021-02543-4 (PMC8613925; doi:10.1186/s13059-021-02543-4)
Supplement: Supplementary file 1 — Additional file 1: Figures S1-13. Supplementary figures [file 13059_2021_2543_MOESM1_ESM.pdf]

Fig. S1

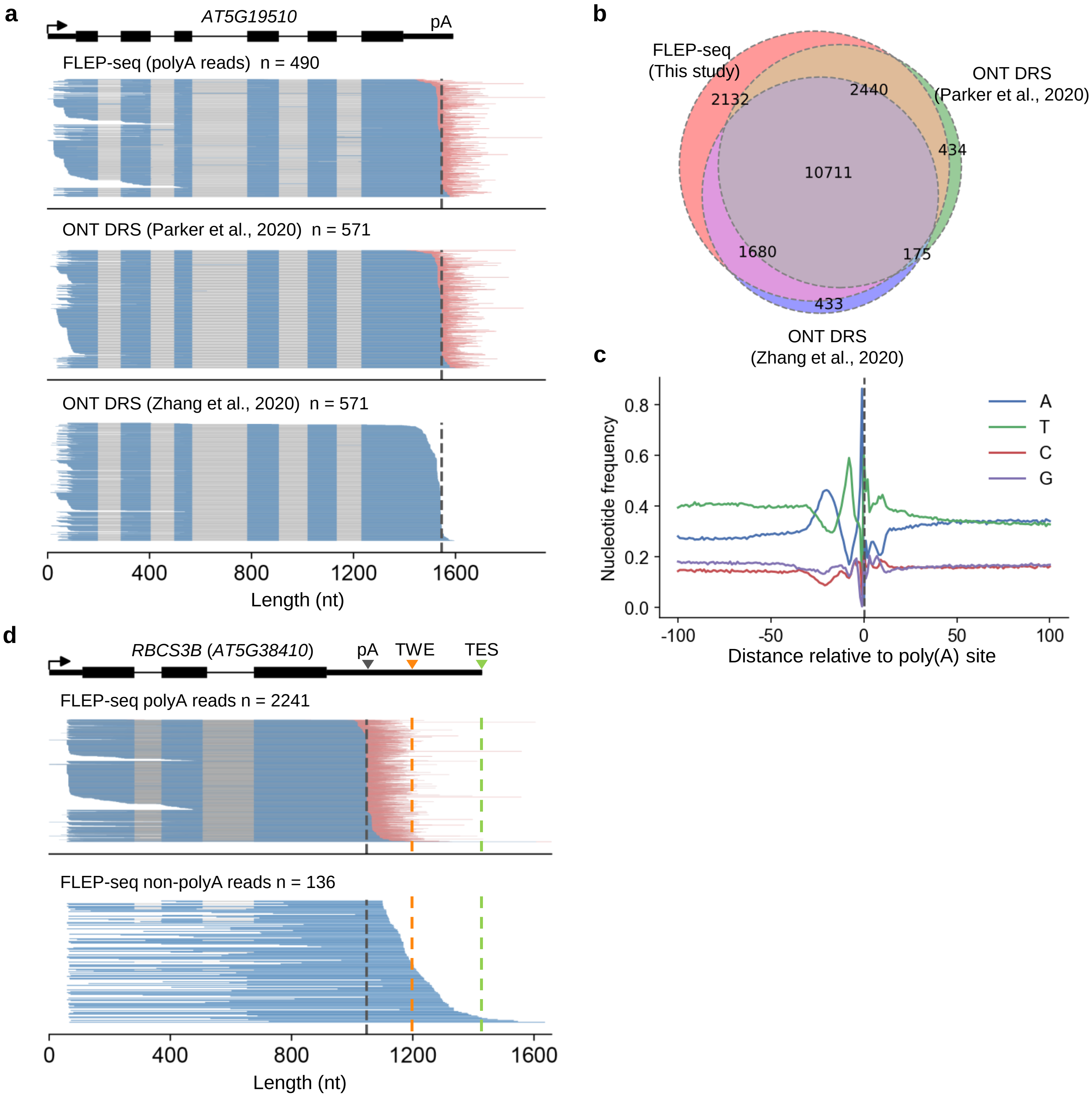

Fig. S1 a. Poly(A)+ reads from FLEP-seq, ONT DRS [30, 40] datasets. The gray black line indicates the poly(A) site (pA). The number of individual reads (n) is indicated. Poly(A) tail length is added to the 3′ end of the read in red (except for Zhang et al. 2020 [40] datasets that provide the FASTQ file after basecalling, but does not includes the raw FAST5 signal file that is required for our nanopolish pipeline). **b.** Venn diagram showing the overlap of poly(A) site clusters identified by Parker et al. 2020 [30], Zhang et al. 2020 [40], and our FLEP-seq. **c.** Nucleotide composition profiles around the poly(A) sites identified by FLEP-seq. **d.** The poly(A) site (pA) and termination window end (TWE) identified by FLEP-seq data are indicated in black and orange dashed lines, respectively. The TES from the Arabidopsis reference annotation is shown as green dashed line.

Fig. S2

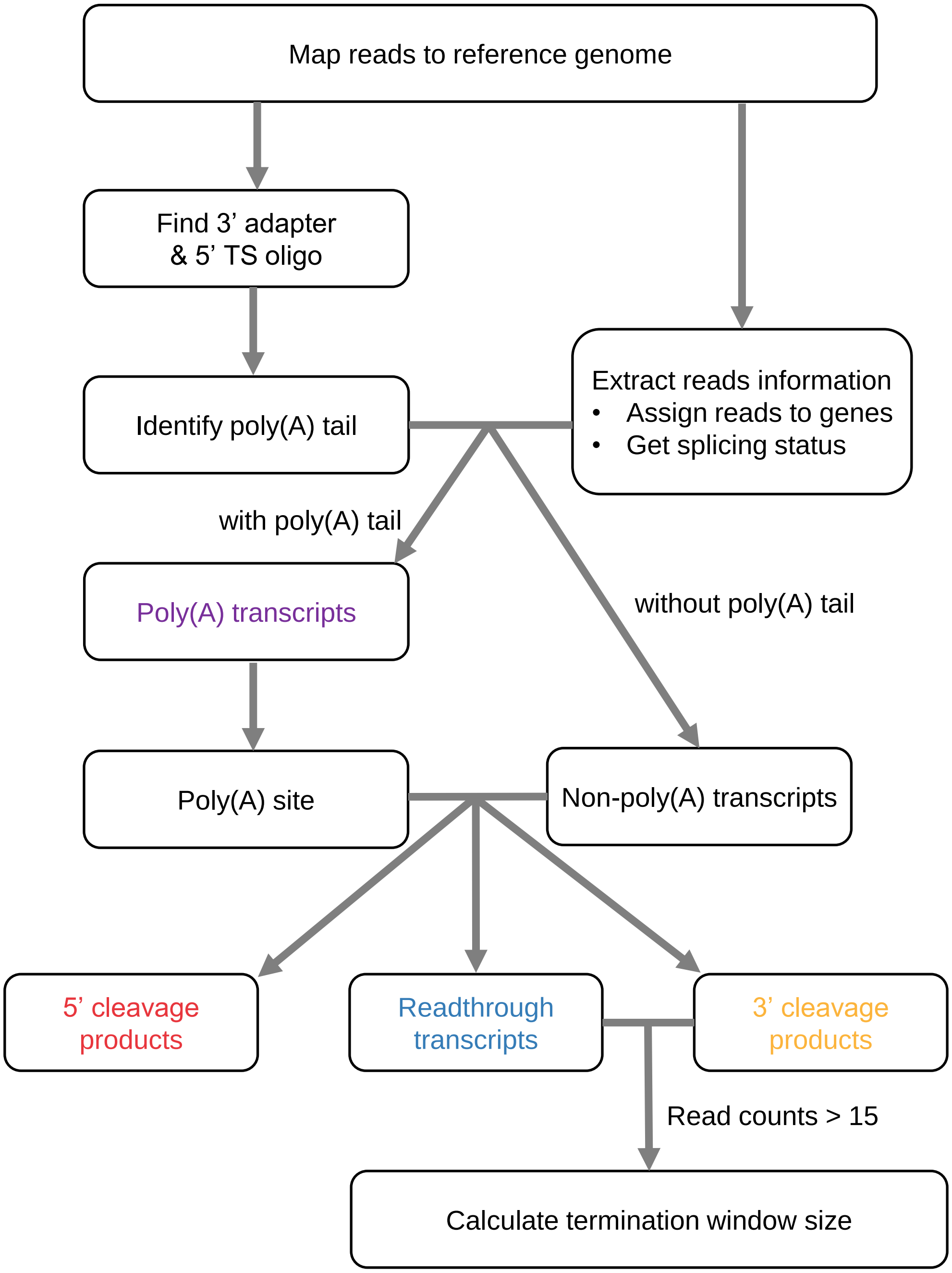

Fig. S2 Schematic workflow for termination landscape analysis. Schematic representation of workflow for handing FLEP-seq data and calculating termination window sizes (see Methods).

Fig. S3

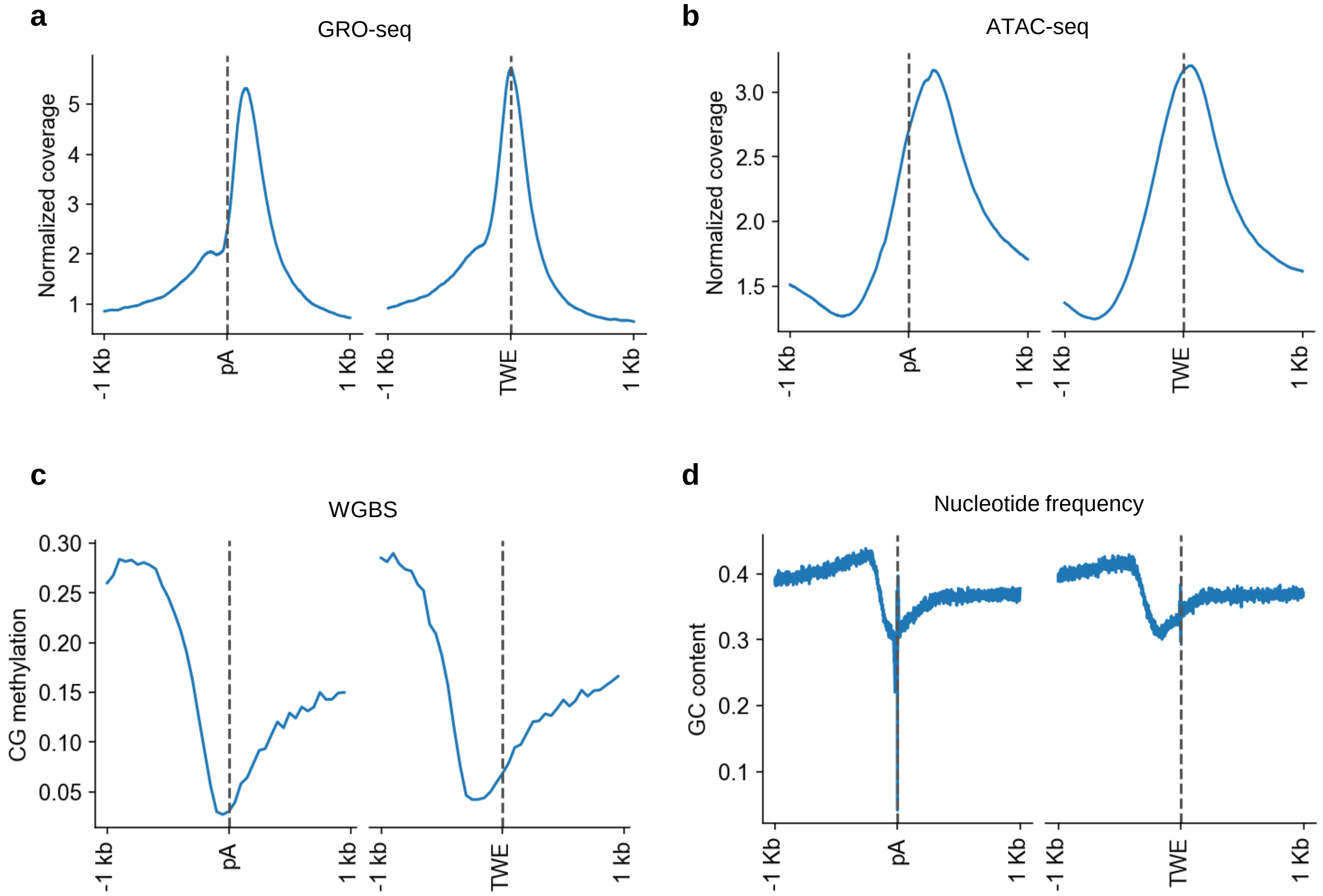

Fig. S3 a, b. Meta-profile of published GRO-seq [43] (**a**), and ATAC-seq [57] (**b**) signal centered around either the poly(A) site (pA) or the termination window end (TWE). **c, d.** CG methylation level (**c**) and GC content (**d**) around the poly(A) site (pA) or the termination window end (TWE).

Fig. S4

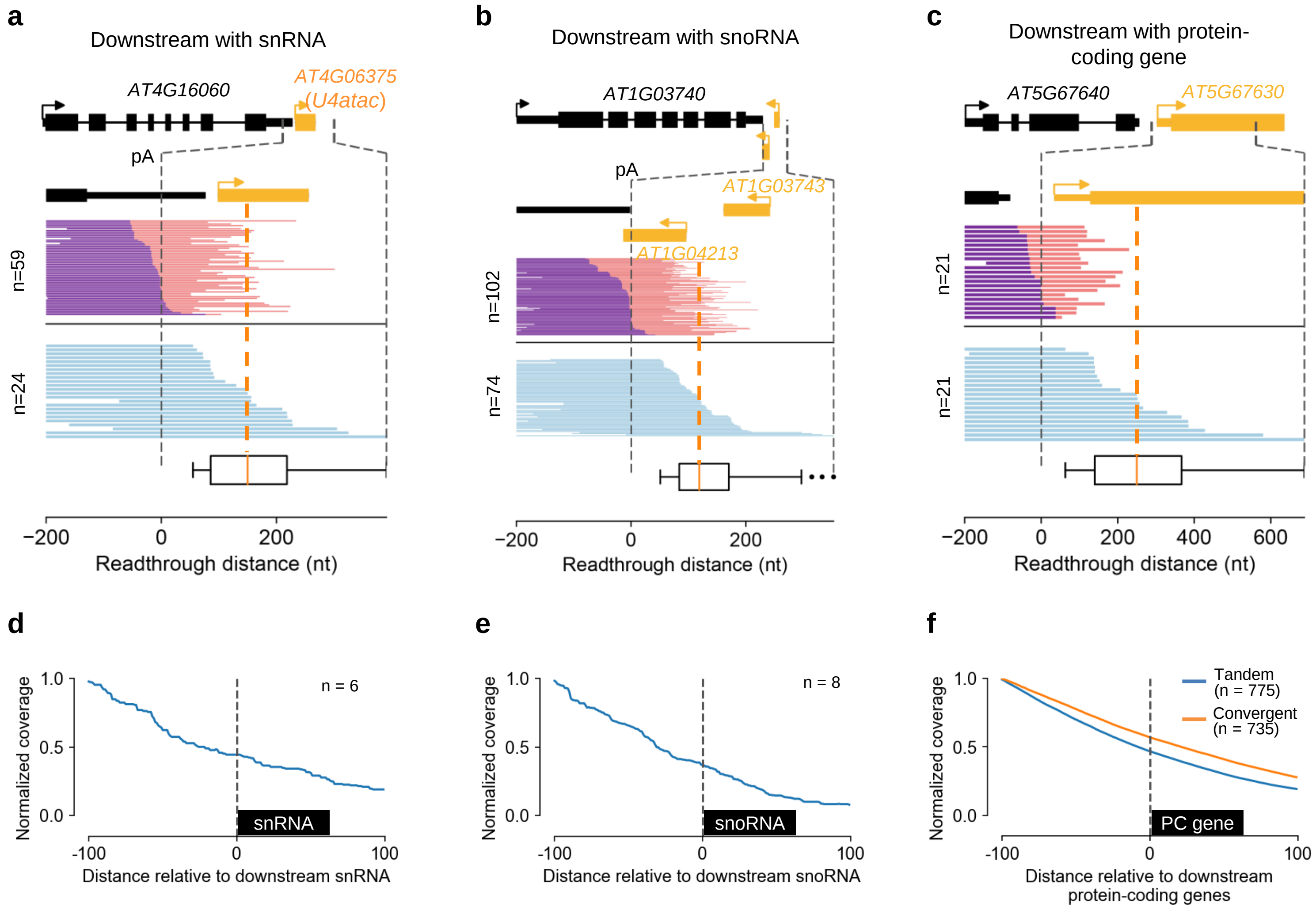

g

Pol II genes with snRNA immediately downstream

Upstream gene	gene strand	Downstream snRNA	snRNA strand	Orientation
AT1G08110	+	AT1G08115	-	convergent
AT3G05700	-	AT3G01715	+	convergent
AT3G56830	-	AT3G56825	-	tandem
AT3G57760	+	AT3G57765	-	convergent
AT4G16060	+	AT4G06375	+	tandem
AT4G35230	-	AT4G09135	+	convergent

h

Pol II genes with snoRNA immediately downstream

Upstream gene	Gene strand	Downstream snoRNA	snoRNA strand	Orientation
AT1G03740	+	AT1G04213, AT1G03743	-	convergent
AT1G06870	+	AT1G04527	-	convergent
AT1G15000	+	AT1G05137	-	convergent
AT2G30200	-	AT2G08375	+	convergent
AT4G13250	-	AT4G13245	+	convergent
AT5G44290	-	AT5G44286	+	convergent
AT5G61440	+	AT5G09000	-	convergent

Fig. S4 Pol II genes that have adjacent non-tRNA genes immediately downstream (distance to downstream gene < 200 nt). a-c. Examples of genes with snRNA, snoRNA, or protein-coding gene immediately downstream, respectively. **d-f.** Meta-profile of Read coverage for genes that have snRNA (**d**), snoRNA (**e**), or protein-coding gene (**f**) immediately downstream, respectively. **g-h.** Pol II genes with snRNA (**g**) or snoRNA (**h**) immediately downstream.

Fig. S5

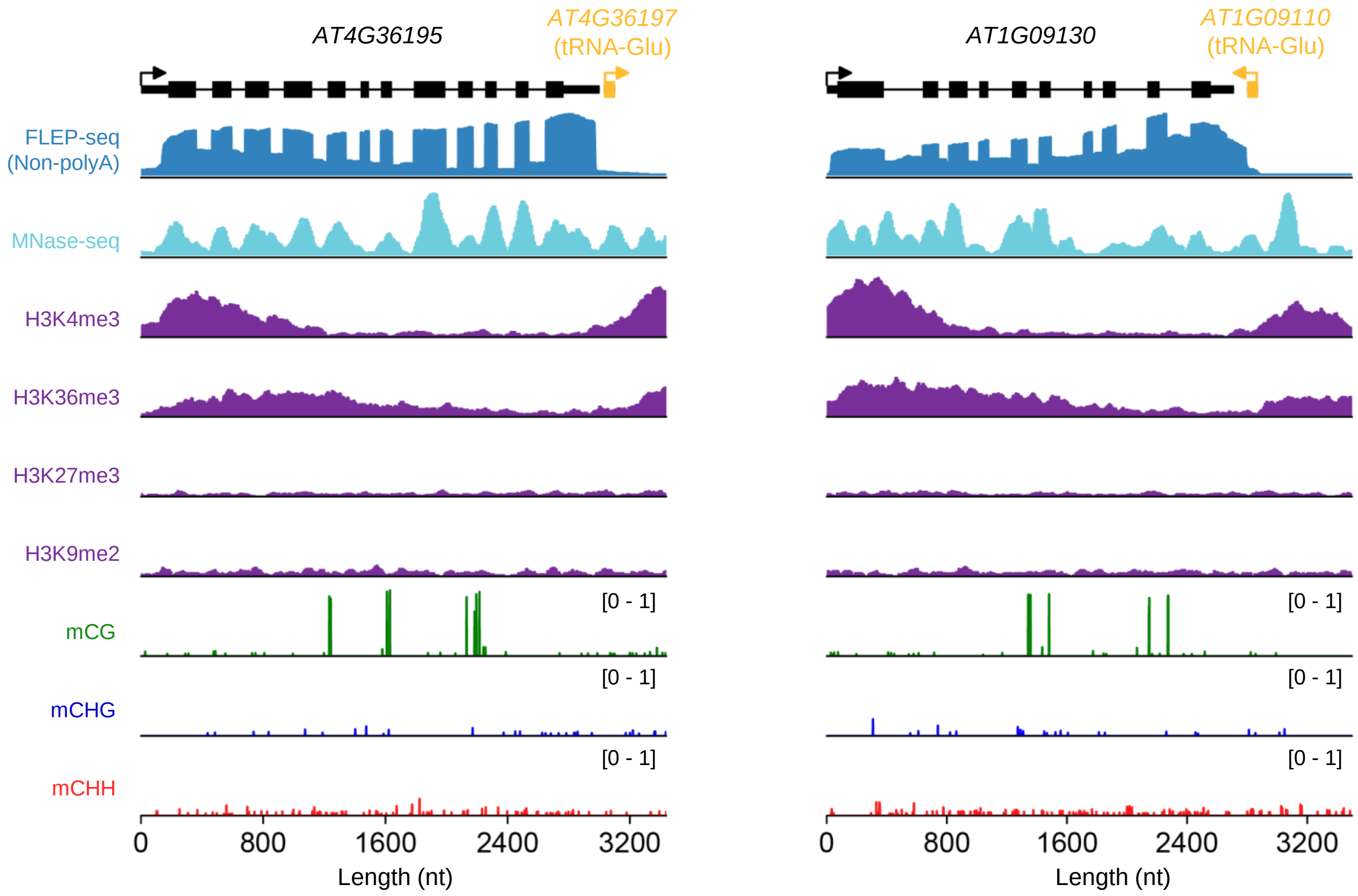

Fig. S5 Local distribution of FLEP-seq, MNase-seq [61], ChIP-seq (H3K4me3, H3K36me3, H3K27me3, and H3K9me2) [62], and DNA methylation for selected genes in **Fig. 4 a, d.**

Fig. S6

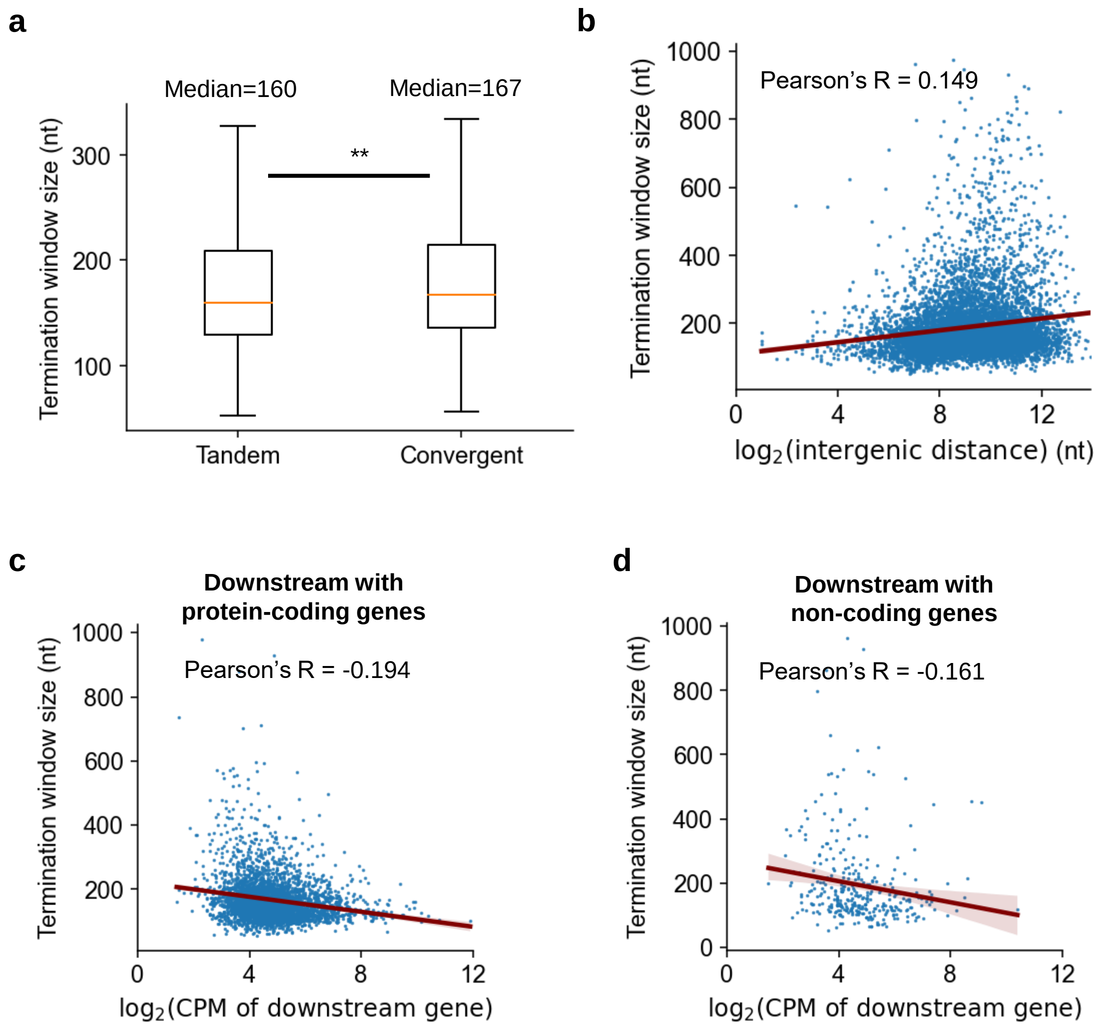

Fig. S6 **a.** Termination window size for genes oriented in different direction. The *p* value was calculated using a Mann–Whitney U test (**, p < 0.001). **b.** Correlation of termination window size with intergenic distance. **c.** Correlation of termination window size with transcription level of downstream protein-coding genes (**c**) and non-coding genes (**d**). Pearson's correlation coefficient is shown.

Fig. S7

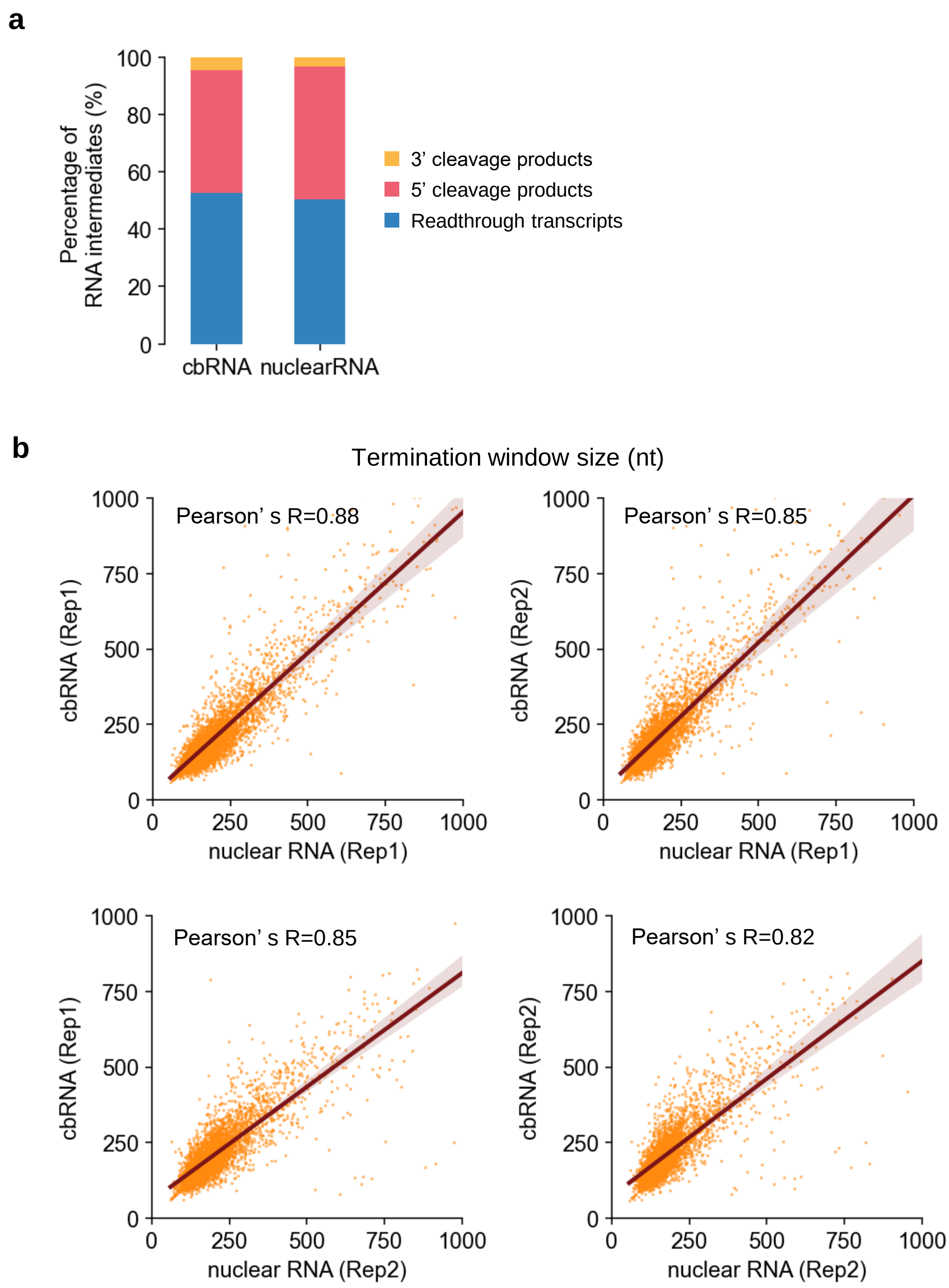

Fig. S7 a. Proportion of non-polyadenylated RNA intermediates in chromatin-bound (cb) RNA and in nuclear RNA data. **b.** Comparison of termination window size identified by cbRNA data and nuclear RNA data**.** The Pearson's correlation coefficient is shown.

Fig. S8

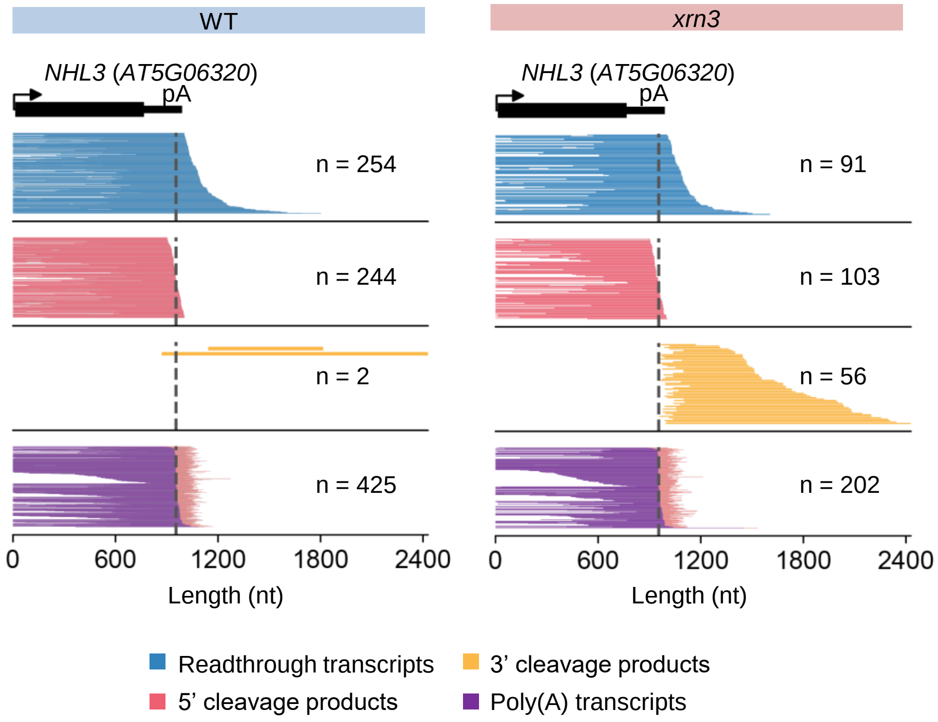

Fig. S8 Examples of genes with increased 3' cleavage products in *atxrn3* mutant. Left panel, wildtype; right panel, *atxrn3* mutant.

Fig. S9

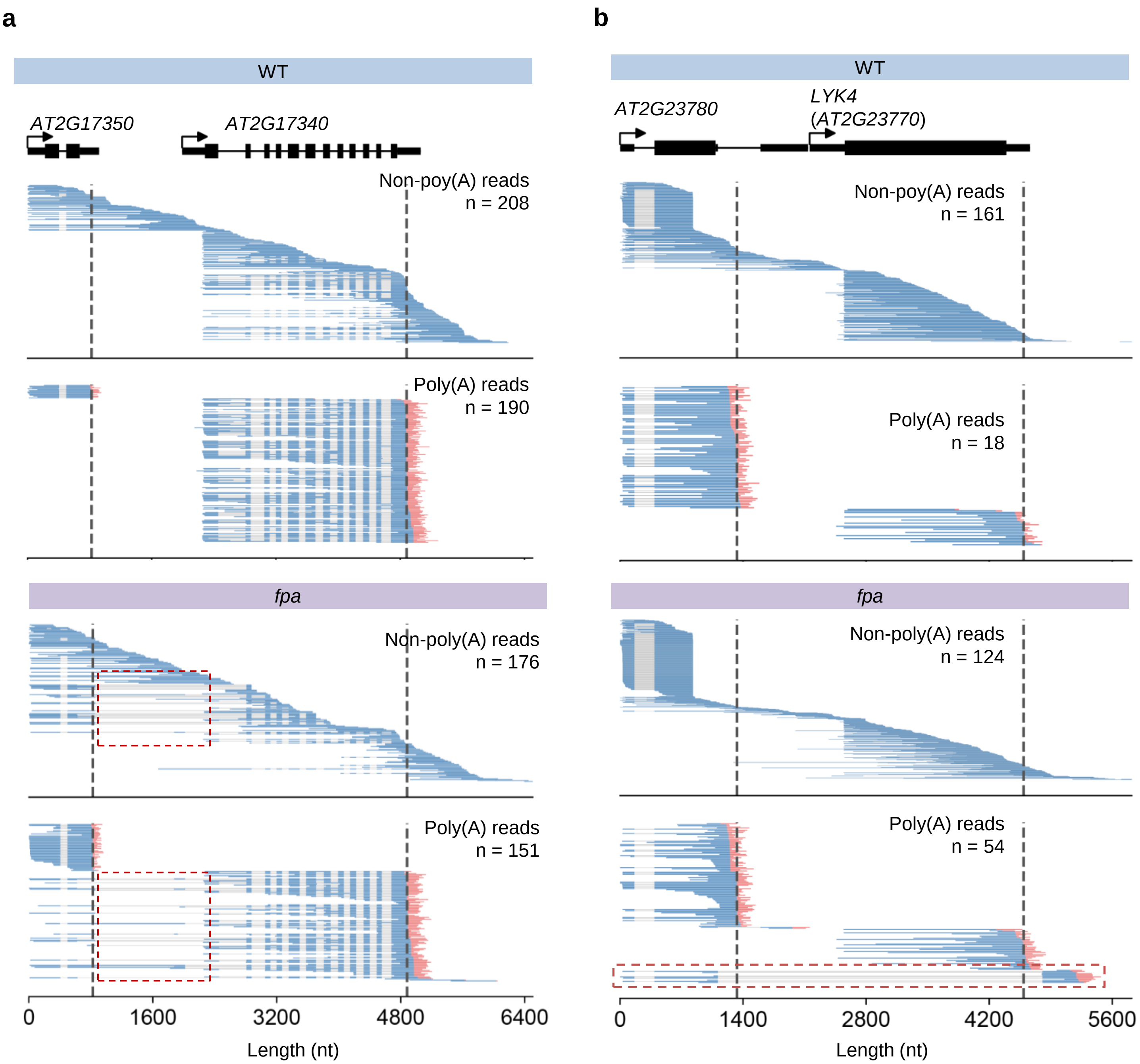

Fig. S9 a-b. Examples of *fpa* mutants in which the gene with readthrough transcript extended into the downstream gene and formed chimeric RNA accompanied by cryptic splicing event. Upper panel, wildtype; lower panel, *fpa* mutant. The black dashed line indicates the poly(A) site. The red dashed box highlighted the cryptic splicing event.

Fig. S10

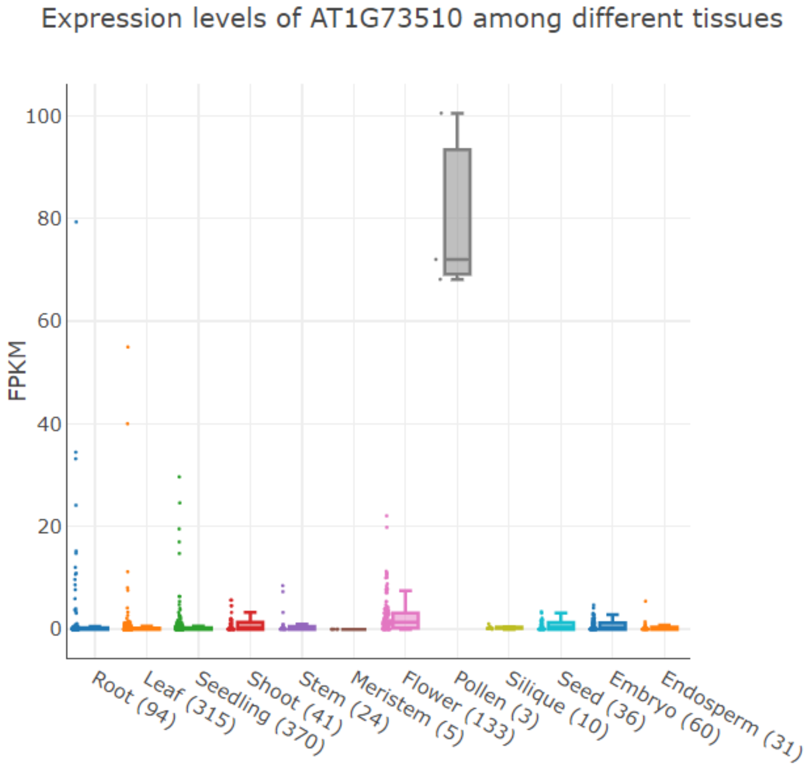

Fig. S10 The expression levels of gene *AT1G73510* among different tissues in Arabidopsis. The expression levels data is obtained from Arabidopsis RNA-seq Database (http://ipf.sustech.edu.cn/pub/athrna/).

Fig. S11

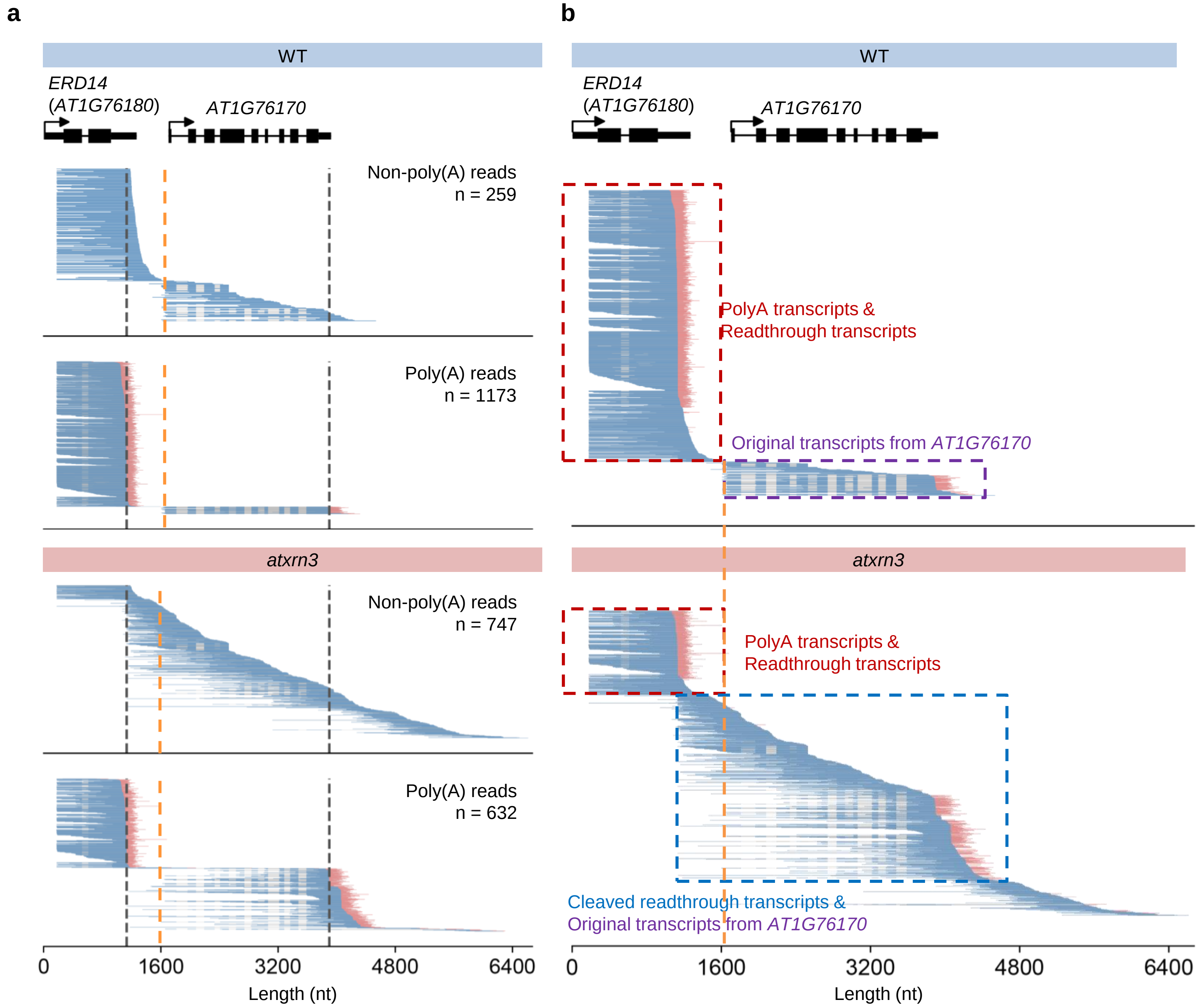

Fig. S11 **a.** Examples of genes with cleaved readthrough reads extended to downstream genes. Upper panel, wildtype; lower panel, *atxrn3* mutant. The black dashed line indicates the poly(A) site. The orange dashed line indicates the TSS of gene *AT1G76170*. **b.** Raw reads aligned to *AT1G76180-AT1G76170* region without classification of read types. Reads are ordered by 3' end position. Each dash block highlights the read types.

Fig. S12

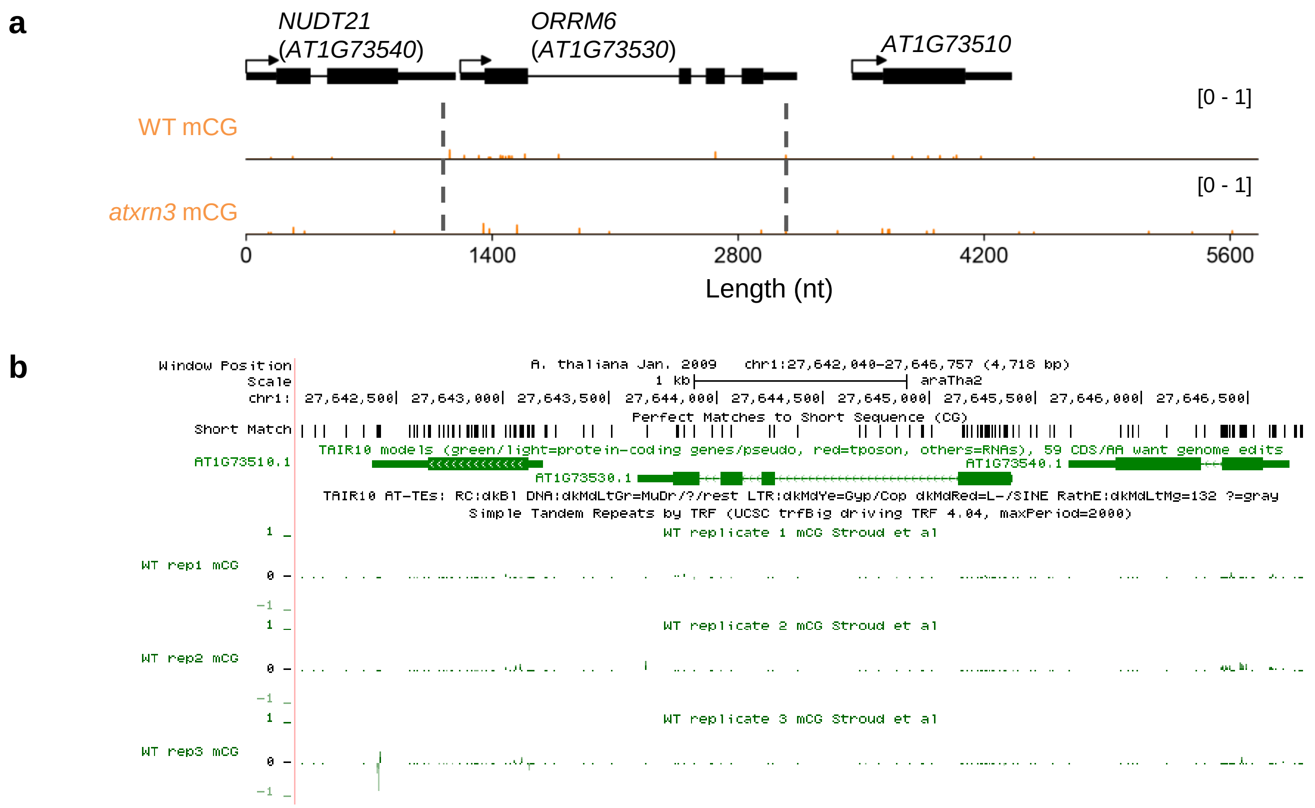

Fig. S12 **a.** CG methylation level around the *AT1G73540-AT1G73510* region in wildtype and *atxrn3* mutant. **b.** CG methylation data from UCSC genome browser around the *AT1G73540-AT1G73510* region [67].

Fig. S13

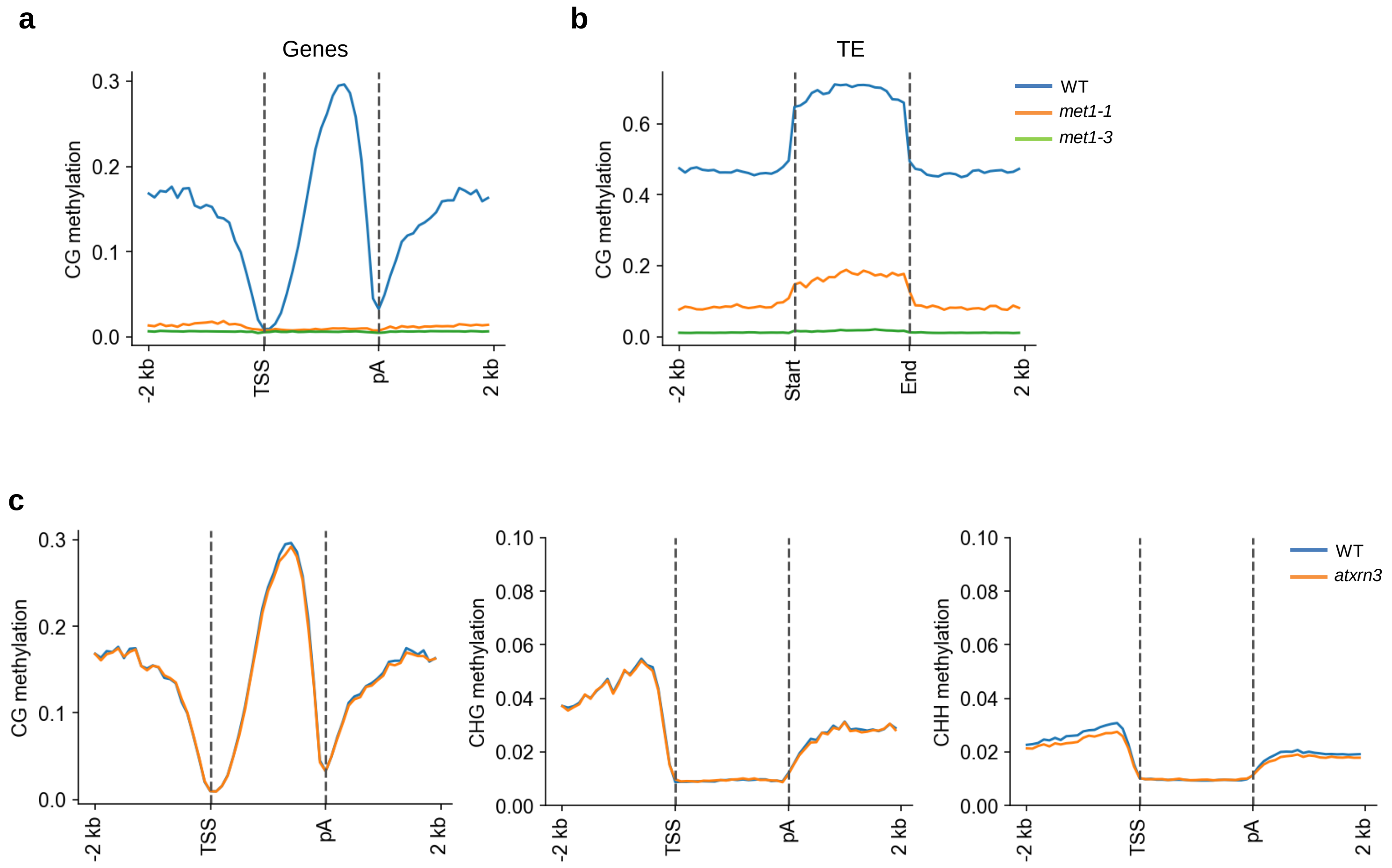

Fig. S13 **a-b.** CG methylation over genes (**a**) and TE (**b**) among wildtype, *met1-1*, and *met1-3* mutant (*met1-3* WGBS data is from Stroud et al., 2013). **c.** CG (left), CHG (middle), and CHH (right) methylation over gene region between wildtype and *atxrn3* mutant.
