# Supplementary material for: Landscape of transcription termination in Arabidopsis revealed by single-molecule nascent RNA sequencing
Source: Genome Biol. 2021 Nov 25;22:322. doi: 10.1186/s13059-021-02543-4 (PMC8613925; doi:10.1186/s13059-021-02543-4)
Supplement: Supplementary file 5 — Additional file 5. Review history. [file 13059_2021_2543_MOESM5_ESM.docx]

Review History

**First round of review**

**Reviewer 1**

**Are you able to assess all statistics in the manuscript, including the appropriateness of statistical tests used?**

No.

**Comments to author:**

Mo, et al. tried to use FLEP-SEQ they invented to profile transcription termination genome-widely. To convince audiences, they also re-analysed previous data related to transcription termination. Furthermore, they also used atxrn3 mutant to confirm the termination window sizes they observed are reliable. In general, it does provide some new information, but lots of problems also exist in current version, and some questions need to be answered.

1. The major sequencing method they used for the presented data did not well described in the method part. Moreover, the method is not an optimal way to profile transcription termination as they mentioned. In the introduction, they ever introduced 4tU-seq and TT-Seq, and acknowledged that they are better strategies to do the work, I wonder why the authors did not use the best strategy to more directly answer the transcription termination question? In FLEP-Seq method, at least two aspects limit its application in profiling transcription termination. 1) ligation efficiency of 3' adapter. As author mentioned in their published protocol, for miRNAs, the 3' adapter ligation efficiency is higher than the long RNA molecules, and people also know that T4 DNA/RNA ligase is low efficiency for RNA ligation, thus the capture RNAs from FLEP-seq may only stands for a portion of nuclear transcriptome. As description in the manuscript that they only profiled less than 10,000 gene loci. In addition, in FLEP-seq, authors also used 11-12 cycles to amplify the cDNAs which may further cause bias to high abundant transcripts, thus decreasing the reliability of data. 2) Normal commercial reverse transcriptase has limitation to amplify longer transcripts (the maximum length is less than 12,000 nt transcripts). While FLEP-Seq used amplified cDNA to evaluate polII transcribed RNA molecules, which may cause inaccurate evaluation of transcription termination of a given gene. E.g. a gene may span several genes and far longer than 12,000 nt, FLEP-seq only can define the transcription termination as its nearest upstream gene, and may not trace to its real original gene. To resolve the problem, ONT DRS should be a good choice.

2. About poly(A) sites (PAS) and transcription termination sites (TTS). In the introduction, authors mentioned the unified model to explain poly(A) signal dependent transcription termination. In the example of fig. 1b, readthrough transcripts reflect those poly(A) signal still not recognized and thus not cleaved transcripts, 3' cleavage products stand for on-going transcribed transcripts after cleavage. According to poly(A) transcripts, there are a few poly(A) transcripts with alternative poly(A) sites far from the defined the poly(A) site. According to the unified model, the transcription termination of these transcripts with alternative poly(A) sites should have their own transcription termination windows, although they come from the same gene loci with those transcripts having a major termination window. The question here, whether poly(A) signal sites and chromatin status around PAS determine transcription termination sites? Could you please check whether the major termination window size mainly locates in low C and G containing genomic regions? Or make a correlation with Steve Jacobsen lab's DNA methylation data in col-0, and see whether there is a correlation between DNA methylation and transcription termination?

3. Mutation of AtXRN3 delays the termination of cleaved readthrough transcripts. In this paragraph, authors found more readthrough transcripts in atxrn3 mutant than in col-0, and thus believe that AtXRN3 play a key role in the termination of cleaved readthrough transcripts. The question here is that when the readthrough transcripts pass through additional poly(A) signal, cleavage and polyadenylation complex will bind to corresponding regions of the transcripts and cleave them, thus, if ATXRN3 just functions as 5' to 3' exonuclease, it should not affect poly(A) site cleavage, thus many At1g73550-at1g73510 fusion poly(A) transcripts in Fig. 6c cannot be well explained with the putative function. Whether AtXRN3 may also affect chromatin status, thus determine poly(A) site selection and readthrough phenomena? Authors should perform DNA methylation assay for those regions around putative poly(A) sites, especially comparing the DNA methylation difference between around putative poly(A) sites and alternative poly(A) sites, e.g. for fig. 6c to compare DNA methylation around At1g73530 poly(A) sites and those around At1g73510 poly(A) sites in mutant and in col-0.

4. In fig. 3b, authors used published BDR1 chip-seq data to show BDR1 signal perfectly located in termination window end as FPA distribution, however, in the original BDR1 paper, BDR1 signal not only enriched in transcription end site (TES), but also enriched in the transcription start sites (TSS), while TES in the literature refers to annotated gene end, which equals to poly(A) sites in the manuscript, why authors got completely different results with the same data? Please explain. In addition, FPA is a trans-acting regulator of poly(A) sites, and it perfectly colocalizes with BDR1, which further confirm that BDR1 may serve as poly(A) site regulator, while poly(A) sites may determine real transcription termination.

5. tRNA genes promote efficient termination of pol II transcription. The conclusion from the current data is not reliable. From the presented data, it only can say that transcription termination may have some correlation with downstream tRNA genes. According to previous literatures, pol II pausing or stalling may caused by chromatin structure, and pausing and stalling of pol II should be a first step for pol II dissociation from DNA templates, pol III occupancy should not be a major reason for the transcription termination of upstream genes since tRNA cannot be transcribed around the clock. One possibility is that chromatin structure around the promoter regions of tRNA genes may block pol II elongation. So, authors can use the published data to explore the chromatin status (e.g. DNA methylation, histone modification, nucleosome positioning, chromosomal conformation capture results, all can be found in Steve Jacobsen lab published data.

6. For poly(A) site comparison in fig. s1a, I wonder why authors used the so-called DRS data (actually it used native RNA as templates and through reverse transcriptase to synthesize fluorescent cDNA for 3' end identification) , ONT DRS data from Arabidopsis have been published in elife and NAR, and both detected poly(A) sites, and both compared the poly(A) site results with the so-called DRS data. Can authors make a comparison with ONT DRS data? 7. Can the author make a gbrowser link for their sequenced reads, like Jacobsen lab (UCLA) did, so that readers can easily find the interested genes from the gbroswer? 8. author should describe their methods in detail, so that readers can reproduce their results according to the provided methods.

**Reviewer 2**

**Are you able to assess all statistics in the manuscript, including the appropriateness of statistical tests used?**

No.

**Comments to author:**

The authors of "Landscape of Transcription Termination in Arabidopsis Revealed by Single-molecule Nascent RNA Sequencing" using single-molecule nascent RNA sequencing to characterize the transcription termination features for Arabidopsis. Overall, the data is well organized and presented and the scientific writing is mostly clear. The method used in this study has been published elsewhere [reference 37, 38]. The authors reanalyzed the published single-molecule nascent RNA sequencing data to profile the transcription termination of Arabidopsis genome. And they also included termination comparison of wildtype and a ribonuclease mutation plant, by sequencing the nucleus RNA. The main novelty of this study is that sequencing nascent RNA using third generation sequencing is more informative than the previous short reads illumina methods, which I think is valid. In addition, they tried to make three main points: 1) the termination windows of gene in Arabidopsis range from 50 nt to over 1000 nt; 2) the termination of protein coding genes followed by tRNA genes was strictly controlled; 3) transcriptional readthrough in atxrn3 might produce spliced and polyadenylated RNA. Although these are interesting findings, some of the points are a little bit exaggerated and lack of solid evidence, new transcription termination mechanisms are expected from such studies. In conclusion, the analyses are preliminary at the moment and the author needs to provide more data to prove that the single-molecule sequencing is more powerful than the short reads method in studying transcription termination.

Major concern,

1. It is already known that the termination window size in plant is smaller than that in mammalian cells, which was indicated by previous studies by illumina nascent RNA sequencing in Arabidopsis (reference 41, 42, 43) and also in other plants (bioRxiv preprint doi: https://doi.org/10.1101/376640). In addition, the termination factors colocalized with 3' Pol II pausing site is also revealed in previous study (reference 52). To get insight in the termination, it is suggested that more mutants to be included using this method, such as CPSF components, BDR1, FPA and ect.

2. It is interesting that tRNA genes might promote efficient termination of Pol II transcription. Is this mechanism specific for tRNA genes? Do the nearby snRNA genes, protein coding genes effect the termination of upstream protein coding genes? The author should check whether the termination window size positively correlate with the intergenic distance, direction, and transcription level of downstream protein coding and/or noncoding genes?

3. Line 199 The xrn3 experiment, the author sequenced the nuclear RNA instead of chromatin bond RNA to capture more 3' cleavage products. Is there any evidence supporting this statement?

4. Line 223 The accumulation of cleaved readthrough transcription which are most likely processed by the cleavage and polyadenylation complex in mutant xrn3 has been proved in previous studies (reference 12). In addition, the author concluded that some cleaved readthrough transcription can yield spliced and polyadenylation mRNA without their own transcription initiation. However, according to the examples in Fig. 6, nearly all spliced transcripts are separated from the upstream gene. Actually, it is hard to distinguish whether these transcripts are the cleaved products of upstream transcriptional readthrough or the products of new initiation. Are there biological replicates for both wildtype and xrn3 plants? More evidence, such as manual verification of certain loci by RT-PCR/seq at both nascent RNA and mRNA levels should be provided. Thus, the conclusion is not rigorous enough.

5. For Figure4-6, other than the certain gene/locus cases, genome wide analysis is expected to make a general point of view. How many genes' termination is regulated by the downstream tRNA or other type of genes? How many genes' termination is xrn3 sensitive and what is the potential determinants? How many genes' cleaved readthrough transcripts enter the downstream genes in xrn3? Minor concerns, 1.Line 84, "Taking DRM2 as an example" 2.Line 90, pNET-seq and plaNET-seq sequence NRPB1 and NRPB2 associate RNA respectively. The pNET-seq is capable to detect different RNA Pol II isoform and thus may distinguish Pol II at certain transcription step. 3.Figure 1b, AT2G3830 should be in italics. 4.Figure 1b, in the pNET-seq track, which antibody was used for this experiment? Ser2P Pol II is supposed to detect the nascent RNA post PAS. 5.Figure 2c, d. Why is the number of genes different? It should be explained in figure legend.

6.Figure3, it will be helpful to compare FLEP-seq with Ser2P and Ser5P-seq data.

7.Figure 5c. The gene name in the brackets should be in italics.

8.Figure 5b. Why there is no sharp peak enriched at pA site for WT as presented in Fig.1f.

9.Figrue 6a, b. The border lines are inconsistent.

10.Line 267. As a scientific name of a species, Arabidopsis thaliana should be in italics.

**Authors Response**

**Point-by-point responses to the reviewers’ comments:**

*Reply: We appreciate the constructive feedback from the two reviewers. We have carefully revised our manuscript according to their helpful comments and suggestions. We have done our best to address the reviewers’ suggestions by adding new data, new analysis, and new discussion points. In particular, we have constructed four new FLEP-seq libraries using nuclear RNA, including the fpa mutant, the met1 mutant, an additional replicate of the atxrn3 mutant, as well as a Col-0 WT control. We also examined the genome-wide DNA methylation status in the atxrn3 mutant and its control via WGBS (Whole-genome Bisulfite Sequencing). In addition, we have toned down or removed a few claims that were previously drawn from weak correlations, and extensively revised the discussion section to include many insightful comments from the reviewers. We are really happy to have had such constructive comments on the paper and feel the manuscript is greatly improved.*

Reviewer #1:

Mo, et al. tried to use FLEP-SEQ they invented to profile transcription termination genome-widely. To convince audiences, they also re-analysed previous data related to transcription termination. Furthermore, they also used atxrn3 mutant to confirm the termination window sizes they observed are reliable. In general, it does provide some new information, but lots of problems also exist in current version, and some questions need to be answered.

*Reply: We thank the reviewer for the constructive feedback.*

1. The major sequencing method they used for the presented data did not well described in the method part. Moreover, the method is not an optimal way to profile transcription termination as they mentioned. In the introduction, they ever introduced 4tU-seq and TT-Seq, and acknowledged that they are better strategies to do the work, I wonder why the authors did not use the best strategy to more directly answer the transcription termination question?

*Reply: We thank the reviewer for these important comments and we apologize for not describing our methods in detail. Since the initial submission of this manuscript, a step-by-step experimental protocol of the FLEP-seq procedure has now been published in Nature Protocols (Long et al., 2021). We have updated this information in the revised text and added a detailed description on how to perform FLEP-seq in the Methods section. In this manuscript, we have provided all the newly developed bioinformatic pipelines for analyzing transcription termination, and uploaded them to GitHub (https://github.com/ZhaiLabSUSTech/Termination_landscape).*

*We fully agree that 4tU-seq/TT-seq are powerful methods for characterizing transcription termination on the Illumina platform. Nevertheless, we would like to clarify that our Nanopore-based single-molecule approach has several unique advantages compared to previous Illumina-based methods:*

*a) There is NO RNA fragmentation step involved in the FLEP-seq procedure, therefore it can capture the full-length RNA intermediates during termination, with information on both their 5’ end and 3’ end positions, as well as the poly(A) tail;*

*b) FLEP-seq can separate the cleaved and uncleaved readthrough RNAs that are previously indistinguishable in the short-read sequencing datasets;*

*c) The single-molecule measurement enables us to quantify the termination window using median readthrough distance, which is less vulnerable to fluctuations in sequencing depth compared to coveragebased assessment from previous Illumina-based methods. With these unique features, we would like to present FLEP-seq as a new method for analyzing transcription termination that is complementary to previous Illumina-based methods.*

*We have also added a new paragraph in discussion to summarize the insightful comments from the reviewer and our responses (L342) –*

*“ Many powerful Illumina-based methods have been applied to characterizing transcription termination, including GRO-seq, NET-seq, 4tU-seq and TT-seq [15, 17, 21, 24]. Nevertheless, our Nanopore-based single-molecule approach has several unique advantages compared to previous Illumina-based methods - a) There is no RNA fragmentation step involved in the FLEP-seq procedure, therefore it can capture the full-length RNA intermediates during termination, with information on both their 5’ end and 3’ end positions, as well as the poly(A) tail; b) FLEP-seq can separate the cleaved and uncleaved readthrough RNAs that are previously indistinguishable in the short-read sequencing datasets; c) The single-molecule measurement enables us to quantify the termination window using median readthrough distance, which is less vulnerable to fluctuations in sequencing depth compared to coverage-based assessment from previous Illumina-based methods. Hence, with these unique features, FLEP-seq could serve as a new method for analyzing transcription termination that is complementary to previous Illumina-based methods. ”*

In FLEP-Seq method, at least two aspects limit its application in profiling transcription termination. 1) ligation efficiency of 3' adapter. As author mentioned in their published protocol, for miRNAs, the 3' adapter ligation efficiency is higher than the long RNA molecules, and people also know that T4 DNA/RNA ligase is low efficiency for RNA ligation, thus the capture RNAs from FLEP-seq may only stands for a portion of nuclear transcriptome. As description in the manuscript that they only profiled less than 10,000 gene loci. In addition, in FLEP-seq, authors also used 11-12 cycles to amplify the cDNAs which may further cause bias to high abundant transcripts, thus decreasing the reliability of data.

*Reply: We thank the reviewer for raising this important point, and indeed adapter ligation is a low-efficiency step that may limit the application of FLEP-seq in ultra-low input situation, such as single-cell analysis. Application of FLEP-seq in bulked sample is not an issue, and in our study we typically use 2 grams of Arabidopsis seedlings as the starting material for each FLEP-seq library. Regarding the number of genes profiled by FLEP-seq, we would like to clarify that we applied a stringent cut-off in our analysis and selected 9,830 genes, each with at least fifteen readthrough reads passed the poly(A) site. The number of gene loci detected by FLEP-seq far exceeds 10,000, and we even detected more genes than the ONT DRS data from polyA+ RNA in Arabidopsis (Parker et al., eLife, 2020).*

2) Normal commercial reverse transcriptase has limitation to amplify longer transcripts (the maximum length is less than 12,000 nt transcripts). While FLEP-Seq used amplified cDNA to evaluate polII transcribed RNA molecules, which may cause inaccurate evaluation of transcription termination of a given gene. E.g. a gene may span several genes and far longer than 12,000 nt, FLEP-seq only can define the transcription termination as its nearest upstream gene, and may not trace to its real original gene. To resolve the problem, ONT DRS should be a good choice.

*Reply: We thank the reviewer for this insightful comment, and we agree with the reviewer that the steps of cDNA amplification and reverse transcription could introduce bias that favors abundant and short transcripts. This is particularly concerning in animal transcripts with much larger introns, and the Churchman group at Harvard Medical School has applied ONT-DRS for nascent RNA sequencing to overcome these issues and analyzed splicing kinetics in human and fly (Drexler et al., Molecular Cell, 2020).*

*However, the current generation of ONT DRS technology has several issues that limit its use in characterizing transcription termination: a) The current ONT DRS kit is only compatible with polyA+ RNAs, thus requires the extra in vitro polyadenylation step to profile nascent RNA that do not have polyA tails (Drexler et al., Molecular Cell, 2020, Drexler et al., Nature Protocols, 2021); b) DRS has much lower yields per run (~1 Million reads per flow cell on MinION) compared to cDNA sequencing (~10 Million reads per flow cell on MinION); c) DRS requires much higher input (500 to 1,000 ng) compared to the as little as 1 ng needed for FLEP-seq.*

*Please see the table below for a brief summary of sequencing statistics that compares data from FLEP-seq and recently published ONT DRS dataset. For example, three DRS MinION runs with human 4sU labeled nascent RNAs only yield 400K reads combined, compared to the over 17 million reads from the two MinION runs of FLEP-seq. The median read length of FLEP-seq is also similar to that of ONT DRS, although maximum read length by ONT DRS is longer. We hope that the future generations of ONT DRS will be able to address these issues so that it can be more easily applied on non-polyA RNAs, and we look forward to updating our method and analysis pipelines along the way.*

*We have updated the discussion to include these insightful points suggested by the reviewer (L370) -*

*“ FLEP-seq approach has its limitations [38]. For example, when RNA is reverse transcribed into cDNA, the integrity of long reads relies on the success of a complete reverse transcription, which favors shorter RNAs. This problem is particularly relevant for animal nascent RNAs with long introns, and recent advancements in Nanopore direct RNA sequencing technology may help to improve this shortcoming [26, 30, 33, 34, 55]. However, the current generation of ONT DRS technology has several issues that limit its use in characterizing transcription termination: a) The current ONT DRS kit is only compatible with polyA+ RNAs, thus requires the extra in vitro polyadenylation step to profile nascent RNA that do not have polyA tails [33, 34]; b) DRS has much lower yields per run (~1 Million reads per flow cell on MinION) compared to cDNA sequencing (~10 Million reads per flow cell on MinION); c) DRS requires much higher input (500 to 1,000 ng) compared to the as little as 1 ng needed for FLEP-seq. We hope that the future generation of ONT DRS will be able to address these issues so that it can be more easily applied on non-polyA RNAs. ”*

2. About poly(A) sites (PAS) and transcription termination sites (TTS). In the introduction, authors mentioned the unified model to explain poly(A) signal dependent transcription termination. In the example of fig. 1b, readthrough transcripts reflect those poly(A) signal still not recognized and thus not cleaved transcripts, 3' cleavage products stand for on-going transcribed transcripts after cleavage. According to poly(A) transcripts, there are a few poly(A) transcripts with alternative poly(A) sites far from the defined the poly(A) site. According to the unified model, the transcription termination of these transcripts with alternative poly(A) sites should have their own transcription termination windows, although they come from the same gene loci with those transcripts having a major termination window. The question here, whether poly(A) signal sites and chromatin status around PAS determine transcription termination sites? Could you please check whether the major termination window size mainly locates in low C and G containing genomic regions? Or make a correlation with Steve Jacobsen lab's DNA methylation data in col-0, and see whether there is a correlation between DNA methylation and transcription termination?

*Reply: We thank the reviewer for this excellent question. In this revision, we have added the analysis of GC content and DNA methylation status near poly(A) signal sites. These results showed that poly(A) sites are featured with low CG methylation and low GC content compared to flanking regions. However, we did not observe obvious change of DNA methylation or GC contents at major site for transcription termination (TWE) (new analysis, Supplementary Figure 3c and 3d). We have added this result to the text (L175) –*

*“ While poly(A) sites are featured with low CG methylation and low GC content compared to flanking regions, we did not observe obvious change of DNA methylation or GC contents at major site for transcription termination (TWE) (Fig. S3c, d). ”*

*To further address the relationship between DNA methylation and termination, we constructed a new FLEPseq library using nuclear RNA from the mutant of DNA methyltransferase MET1 (Kankel et al., Genetics, 2003), in which virtually all CG methylation is lost. Our result showed that the size of termination windows in the met1-1 mutant is similar to that in WT Col-0 (new data, Figure 5d), and the distribution for 3’ ends of readthrough RNAs is also unaffected in met1-1 (new data, Figure 5c). These results suggest that DNA methylation status is not directly correlated with transcription termination. We have revised the main text to add this important information on the correlation between DNA methylation and transcription termination (L276) -*

*“ In the met1-1 mutant of DNA methyltransferase MET1 [62], in which virtually all CG methylation is lost, the distribution for 3’ ends of readthrough RNAs is also unaffected in met1-1 (Fig. 5c), and the size of termination windows is similar to that in wildtype Col-0 (Fig. 5d). These results suggest that DNA methylation status is not directly correlated with transcription termination. ”*

3. Mutation of AtXRN3 delays the termination of cleaved readthrough transcripts. In this paragraph, authors found more readthrough transcripts in atxrn3 mutant than in col-0, and thus believe that AtXRN3 play a key role in the termination of cleaved readthrough transcripts. The question here is that when the readthrough transcripts pass through additional poly(A) signal, cleavage and polyadenylation complex will bind to corresponding regions of the transcripts and cleave them, thus, if ATXRN3 just functions as 5' to 3' exonuclease, it should not affect poly(A) site cleavage, thus many At1g73530-at1g73510 fusion poly(A) transcripts in Fig. 6c cannot be well explained with the putative function. Whether AtXRN3 may also affect chromatin status, thus determine poly(A) site selection and readthrough phenomena? Authors should perform DNA methylation assay for those regions around putative poly(A) sites, especially comparing the DNA methylation difference between around putative poly(A) sites and alternative poly(A) sites, e.g. for fig. 6c to compare DNA methylation around At1g73530 poly(A) sites and those around At1g73510 poly(A) sites in mutant and in col-0.

*Reply: We agree with the reviewer that accumulation of the AT1G73530-AT1G73510 fusion poly(A) transcripts cannot be solely explained by the loss of 5' to 3' exonuclease activity. To check if DNA methylation status is altered at poly(A) sites in the atxrn3 mutant, we performed Whole Genome Bisulfite Sequencing (WGBS) in seedlings of atxrn3 and corresponding Col-0 control (new data). At the AT1G73530- AT1G73510 loci, there is little CG methylation in the wildtype, and it remains mostly unmethylated in the atxrn3 mutant (new data, Supplementary Figure 12a). This is consistent with previous DNA methylation profiling of multiple wildtype Arabidopsis libraries from the Jacobsen group (new analysis, Supplementary Figure 12b). In addition, we found the overall DNA methylation pattern at genic regions remains unchanged in the atxrn3 mutant compared to WT (new data, Supplemental Figure 12c). As the reviewer suggested, we also compared the DNA methylation status around putative poly(A) sites and alternative poly(A) sites for those genes carrying both types of ploy(A) sites, and there is no obvious difference between these two types in either the wildtype or the atxrn3 mutant (new analysis, Supplementary 12d). In this revision, we have toned down the claim on the role of AtXRN3 in termination, and added the possibility that other factors, including chromatin status and DNA modification, could contribute to the accumulated readthrough transcripts in the atxrn3 mutant (L315) -*

*“ However, the AT1G73530-AT1G73510 fusion poly(A) transcripts cannot be solely explained by the loss of 5’ to 3’ exonuclease activity as AtXRN3 should not affect the cleavage at the poly(A) site. It is possible that AtXRN3 may affect chromatin status that in turn determines poly(A) site selection and the readthrough phenomena. To check if DNA methylation status is altered at poly(A) sites in the atxrn3 mutant, we performed Whole Genome Bisulfite Sequencing (WGBS) in seedlings of atxrn3 and corresponding Col-0 control. At the AT1G73530-AT1G73510 loci, there is little CG methylation in the wildtype, and it remains mostly unmethylated in the atxrn3 mutant (Fig. S12a). This is consistent with previous DNA methylation profiling of multiple wildtype Arabidopsis libraries from the Jacobsen group [63] (Fig. S12b). In addition, we found the overall DNA methylation pattern at genic regions remains unchanged in the atxrn3 mutant compared to WT (Fig. S12c). We also compared the DNA methylation status around putative poly(A) sites and alternative poly(A) sites for those genes carrying both types of ploy(A) sites, and there is no obvious difference between these two types in either the wildtype or the atxrn3 mutant (Fig. S12d). Therefore, besides exonuclease activity of AtXRN3, other factors such as chromatin status and modification could also contribute to the accumulated readthrough transcripts in the atxrn3 mutant. ”*

4. In fig. 3b, authors used published BDR1 chip-seq data to show BDR1 signal perfectly located in termination window end as FPA distribution, however, in the original BDR1 paper, BDR1 signal not only enriched in transcription end site (TES), but also enriched in the transcription start sites (TSS), while TES in the literature refers to annotated gene end, which equals to poly(A) sites in the manuscript, why authors got completely different results with the same data? Please explain. In addition, FPA is a trans-acting regulator of poly(A) sites, and it perfectly colocalizes with BDR1, which further confirm that BDR1 may serve as poly(A) site regulator, while poly(A) sites may determine real transcription termination.

*Reply: We thank the reviewers for this comment, and we apologize for not describing this clearly in the previous version.*

*In the original BDR1 paper, the authors used the TAIR annotated end position of a gene as “TES” and found enrichment of BDR1 around TES. We want to clarify that their TES is NOT equal to the poly(A) sites we used. The TES positions in TAIR are based on the reconstructed transcript assembly using a collection of Illumina RNA-seq data that comprised of 113 RNA-seq datasets from eleven different tissues (Cheng et al., The Plant Journal, 2017). The figure below is an example of poly(A) site being different from the TAIR annotated TES and the TWE (Termination Window End) defined by our FLEP-seq data (new analysis, Supplementary Figure 1d). We hope our data will be helpful to further refine the annotation of TES in future versions of the Arabidopsis genome annotation.*

*We also tried to plot the ChIP-seq data of BDR1 and FPA at poly(A) site, TES, and TWE. The peaks of BDR1 and FPA are both centered at TWE.*

*We realize that the definition of polyA site, TES, and TWE can be confusing to the readers, and we have added a paragraph and a supplemental figure to the main text for clarification (L180) -*

*“Previous study demonstrated that BDR proteins prefer to reside at gene borders, in both transcription start sites (TSS) and transcription end site (TES) [53]. It is worth to clarify that poly(A) site, TES, and TWE are different from each other – poly(A) site is where cleavage and polyadenylation occurs; TES in TAIR are defined based on the reconstructed transcript assembly using a collection of Illumina RNA-seq [19]; and TWE is the median readthrough length defined by FLEP-seq data (Fig. S1d).”*

5. tRNA genes promote efficient termination of pol II transcription. The conclusion from the current data is not reliable. From the presented data, it only can say that transcription termination may have some correlation with downstream tRNA genes. According to previous literatures, pol II pausing or stalling may be caused by chromatin structure, and pausing and stalling of pol II should be a first step for pol II dissociation from DNA templates, pol III occupancy should not be a major reason for the transcription termination of upstream genes since tRNA cannot be transcribed around the clock. One possibility is that chromatin structure around the promoter regions of tRNA genes may block pol II elongation. So, authors can use the published data to explore the chromatin status (e.g. DNA methylation, histone modification, nucleosome positioning, chromosomal conformation capture results, all can be found in Steve Jacobsen lab published data.

*Reply: We agree with the reviewer that many factors other than Pol III occupancy could contribute to the efficient termination we observed upstream of tRNA genes. We have significantly tuned down the claim on tRNA related termination in the abstract (L6-8) –*

*The previous “We also discovered a termination mechanism shaped by the downstream tRNA genes, suggesting that elongating Pol II can be expelled by Pol III activity.” is now changed to “We also observed efficient termination before downstream tRNA genes, suggesting that chromatin structure around the promoter region of tRNA genes may block pol II elongation.”*

*We thank the reviewer for the suggestion to check the chromatin status around tRNA genes. In this revision, we have analyzed published data of MNase-seq, ChIP-seq (H3K4me3, H3K36me3, H3K27me3, H3K9me2), as well as the CG, CHG, and CHH DNA methylation level from our newly added WGBS data to check for chromatin status at the two tRNA loci from Figure 4 (new analysis, Supplementary Figure 5). We did not observe an obvious enrichment or depletion of the common epigenetic marks around the tRNA promoters, and we hope future studies on factors that bind to the promoter of tRNA genes could help explain its role in blocking Pol II transcription. We also found a previous report in Leishmania major that also found tRNA gene can serve as terminator for Pol II transcription in Trypanosomatida (Martínez-Calvillo et al., Eukaryotic Cell, 2004). We have updated the main text to include the insightful comments from the reviewer and our new analysis (L204) –*

*“Moreover, previous literature has shown that Pol II pausing or stalling can be influenced by chromatin structure [2]. Therefore, it is possible that the termination of Pol II elongation at -60 nt is caused by a unique chromatin status at the tRNA gene promoter. To test this scenario, we analyzed published data of MNaseseq, ChIP-seq (H3K4me3, H3K36me3, H3K27me3, H3K9me2), as well as the CG, CHG, and CHH DNA methylation level to check the chromatin status at the two tRNA loci showed in Fig. 4, including nucleosome positioning, histone modification, and DNA methylation (Fig. S5). We did not observe an obvious enrichment or depletion of the common epigenetic marks around the tRNA promoters. Future studies on the factors that bind to the promoter of tRNA gene may help to explain its role in blocking Pol II transcription. Besides Arabidopsis, a previous report in Leishmania major also found tRNA gene can serve as terminator for Pol II transcription in the trypanosomatida [60].”*

*As the reviewer suggested, we also analyzed the Dpn II Hi-C data (Wang et al., Genome Res., 2015) to check the chromosomal conformation capture result at tRNA genes. Unfortunately, due to the short length of tRNA genes and the lack of Dpn II digestion sites nearby, we could not observe any clear pattern. The Figure for response below is an example of Hi-C data near a tRNA gene in the middle.*

6. For poly(A) site comparison in fig. s1a, I wonder why authors used the so-called DRS data (actually it used native RNA as templates and through reverse transcriptase to synthesize fluorescent cDNA for 3' end identification) , ONT DRS data from Arabidopsis have been published in elife and NAR, and both detected poly(A) sites, and both compared the poly(A) site results with the so-called DRS data. Can authors make a comparison with ONT DRS data?

*Reply: We fully agree with the reviewer that the ONT DRS data would be a better source for poly(A) site identification. In this revision, we analyzed the ONT DRS data from Parker et al., eLife, 2020 and Zhang et al., Nucleic Acids Research, 2020. The polyA sites obtained from these two datasets are highly consistent with the ones from FLEP-seq data (new analysis, updated Supplementary Figure 1a and 1b).*

7. Can the author make a gbrowser link for their sequenced reads, like Jacobsen lab (UCLA) did, so that readers can easily find the interested genes from the gbroswer?

*Reply: We thank the reviewer for this excellent suggestion. We have created a JBrowse website for hosting the FLEP-seq data (https://zhailab-sustech.github.io/jbrowse2/). Readers can search for their gene of interest and quickly obtain the sequence information. We also added the positions of poly(A) site, TES and TWE as annotation.*

8. author should describe their methods in detail, so that readers can reproduce their results according to the provided methods.

*Reply: Our apology for insufficient detail on the FLEP-seq method. Since the initial submission of this manuscript, a step-by-step experimental protocol of the FLEP-seq procedure has now been published in Nature Protocols (Long et al., 2021). We have updated this information in the revised text and added a detailed description of how to perform FLEP-seq in the Methods section (L399) –*

*“****Nuclear RNA extraction and FLEP-seq library construction***

*Nuclear RNA extraction is performed according to the previously reported method [37, 38]. In brief, 2 g of seedlings were ground with liquid nitrogen and transferred to ice-cold RNase/DNase-free 50 ml tube with 10 ml Honda buffer (0.44 M sucrose, 1.25% (w/v) Ficoll, 2.5% (w/v) dextran T40, 20 mM HEPES-KOH pH7.4, 10 mM MgCl2, 0.5% (w/v) Triton X-100, 1 mM dithiothreitol (DTT), 1× protease inhibitor (Roche) and 100 ng μl−1 tRNA). The samples were homogenized and filtered through Miracloth, and the remaining samples on filter were washed with another 10 ml Honda buffer. After centrifuged at 2,000 g for 5 min at 4 °C, the nuclear pellets were resuspended and washed twice with 15 ml Honda buffer. The pellets were transferred to a 1.5 ml RNase/DNase-free microcentrifuge tube in 1 ml Honda buffer, and centrifuged at 8,000 g for 1 min at 4 °C. Then the supernatant was completely removed. For RNA extraction, the nuclei pellet was resuspended with 1 ml TRIzol and vortexed to mix thoroughly. After 10 min incubation at room temperature, 0.2× volume of chloroform was added, mixed, and incubated at room temperature for 5 min. The mixture was centrifuged at 14,000 g for 10 min at 4 °C. The supernatant was collected and added with one volume of 100% (w/v) ethanol. The RNA extraction was performed according to the manufacturer’s instructions (ZYMO, R2070). RNA concentration was measured by Nanodrop and Qubit 3.0 fluorometer with Equalbit RNA HS assay kit (Vazyme, EQ211-01).*

*At least 2 μg nuclear RNA was used for FLEP-seq library preparation. Ribosomal RNA (rRNA) was depleted using riboPOOL kit (siTOOLs Biotech) following the manufacturer’s instructions. After purification with the ZYMO RNA Clean & Concentrator-5 kit ZYMO, R1013), the nuclear RNA was mixed with 50 pmol 3’ adapter (5’-rAppCTGTAGGCACCATCAAT–NH2-3’, NEB, S1315S) at 65 ℃ for 5 min, and then placed on ice for more than 1 min. 2 μl 10× T4 RNA ligase reaction buffer (NEB, M0242), 10 μl 50% PEG 8000 (NEB, M0242), 1 μl 40 U μl-1 Murine Rnase Inhibitor (Vazyme, R301-03 ) and 1 μl T4 RNA ligase 2, truncated K227Q (NEB, M0242) were added to the RNA and mixed thoroughly. The ligation reaction was performed at 16 ℃ for 10 h. After that, the RNA was purified and concentrated to 6 μl using the ZYMO RNA Clean & Concentrator kit (ZYMO, R1013). Reverse transcription and template-switching were performed using the SMARTer PCR cDNA Synthesis Kit (Takara, 634926) with minor modification. The SMART CDS Primer II A was replaced by the custom 3’ cDNA RT primer (5’- AAGCAGTGGTATCAACGCAGAGTACATTGATGGTGCCTACAG-3’), which is complementary to the 3’ adapter sequence. To minimize the PCR bias resulting from over-amplification, PCR cycle optimization was performed to determine the best cycle number for cDNA amplification. The cDNA for Nanopore library construction was amplified using the optimized cycle number and purified twice with the VAHTS DNA Clean Beads (Vazyme, N411). DNA concentration and quality were measured by Qubit 3.0 and Agilent 2100 Bioanalyzer. Then, 200 fmol DNA was used to construct the Nanopore library using the Ligation Sequencing Kit (SQK-LSK109, Oxford Nanopore Technologies) according to the instruction. Libraries were loaded onto R9.4 flow cell (Oxford Nanopore Technologies) and sequenced on MinION device for ~48 hours. ”*

Reviewer #1:

The authors of "Landscape of Transcription Termination in Arabidopsis Revealed by Single-molecule Nascent RNA Sequencing" using single-molecule nascent RNA sequencing to characterize the transcription termination features for Arabidopsis. Overall, the data is well organized and presented and the scientific writing is mostly clear. The method used in this study has been published elsewhere [reference 37, 38]. The authors reanalyzed the published singlemolecule nascent RNA sequencing data to profile the transcription termination of Arabidopsis genome. And they also included termination comparison of wildtype and a ribonuclease mutation plant, by sequencing the nucleus RNA. The main novelty of this study is that sequencing nascent RNA using third generation sequencing is more informative than the previous short reads illumina methods, which I think is valid. In addition, they tried to make three main points: 1) the termination windows of gene in Arabidopsis range from 50 nt to over 1000 nt; 2) the termination of protein coding genes followed by tRNA genes was strictly controlled; 3) transcriptional readthrough in atxrn3 might produce spliced and polyadenylated RNA. Although these are interesting findings, some of the points are a little bit exaggerated and lack of solid evidence, new transcription termination mechanisms are expected from such studies. In conclusion, the analyses are preliminary at the moment and the author needs to provide more data to prove that the single molecule sequencing is more powerful than the short reads method in studying transcription termination.

*Reply: We thank the reviewer for the highly constructive feedback. We have revised the study by including new data and new analyses to directly address the reviewers’ concerns as described below.*

Major concern,

1. It is already known that the termination window size in plant is smaller than that in mammalian cells, which was indicated by previous studies by illumina nascent RNA sequencing in Arabidopsis (reference 41, 42, 43) and also in other plants (bioRxiv preprint doi: https://doi.org/10.1101/376640). In addition, the termination factors colocalized with 3' Pol II pausing site is also revealed in previous study (reference 52). To get insight in the termination, it is suggested that more mutants to be included using this method, such as CPSF components, BDR1, FPA and ect.

*Reply: We thank the reviewer for this very important suggestion, and our apology for having missed the paper on GRO/PRO-seq analysis in cassava and maize. We have updated our text to include this very relevant reference.*

*We appreciate the reviewer's suggestion on applying FLEP-seq to studying more mutants that may affect termination. In this revision, we have characterized two additional mutants by FLEP-seq, fpa and met1. FPA is a component of the 3’ end processing complex, and MET1 is the key DNA methyltransferase in Arabidopsis.*

*The fpa mutant FLEP-seq data showed a global impact on termination window size and a prolonged 3’end distribution compared to WT (new data, Figure 5c and 5d), consistent with its previously reported function as a termination factor (Duc et al., PLoS Genet., 2013; Parker et al., eLife, 2021). We also found some interesting cases that support the previous report of chimeric transcripts and cryptic splicing occurring in the fpa mutant (Duc et al., PLoS Genet., 2013; Parker et al., eLife, 2021). For example, some readthrough transcripts in the fpa mutant can extend into downstream genes to form chimeric RNAs accompanied by the cryptic splicing event (new data, Supplementary Figure S9a). Cryptic splicing event in the fpa mutant can even result in excision of the entire gene in the middle from the readthrough transcript that spans multiple genes (new data, Supplementary Figure S9b).*

*We also constructed a new FLEP-seq library from the mutant of DNA methyltransferase MET1 (Kankel et al., Genetics, 2003), in which virtually all CG methylation is lost. Our result showed that the size of termination windows in the met1-1 mutant is similar to that in WT Col-0 (new data, Figure 5d), and the distribution for 3’ ends of readthrough RNAs is also unaffected in met1-1 (new data, Figure 5c). These results suggest that DNA methylation status is not directly correlated with transcription termination.*

*We have revised the abstract and the main text to add the characterization of fpa and met1 mutants by FLEP-seq (L267) –*

*“In addition to atxrn3, we also characterized the fpa and met1 mutants by FLEP-seq. FPA is a component of the 3’ end processing complex, and MET1 is the key DNA methyltransferase in Arabidopsis [62]. The fpa mutant FLEP-seq data showed a global impact on termination window size and a prolonged 3’end distribution compared to WT (Fig. 5c-d), consistent with its previously reported function as a termination factor [12, 55]. We also found some interesting cases that support the previous report of chimeric transcripts and cryptic splicing occurring in the fpa mutant [12, 55]. For example, some readthrough transcripts in the fpa mutant can extend into downstream genes to form chimeric RNAs accompanied by the cryptic splicing event (Fig. S9a). Cryptic splicing event in the fpa mutant can even result in excision of the entire gene in the middle from the readthrough transcript that spans multiple genes (Fig. S9b). In the met1-1 mutant of DNA methyltransferase MET1 [62], in which virtually all CG methylation is lost, the distribution for 3’ ends of readthrough RNAs is also unaffected in met1-1 (Fig. 5c), and the size of termination windows is similar to that in wildtype Col-0 (Fig. 5d). These results suggest that DNA methylation status is not directly correlated with transcription termination.”*

*We are in contact with Professor Scott Michaels group for obtaining the bdr1/2/3 triple mutant, and we are currently collaborating with research groups to generate high-order mutants of CPSF components using multiplexed CRISPR-Cas9 editing due to its large number of family members in plants. In the future, we plan to carry out FLEP-seq in these mutants suggested by the reviewer and continue investigating the mechanism for transcription termination in plants.*

2. It is interesting that tRNA genes might promote efficient termination of Pol II transcription. Is this mechanism specific for tRNA genes? Do the nearby snRNA genes, protein coding genes effect the termination of upstream protein coding genes? The author should check whether the termination window size positively correlate with the intergenic distance, direction, and transcription level of downstream protein coding and/or noncoding genes?

*Reply: We thank the reviewer for this suggestion. In this revision, we checked if snRNA, snoRNA, and protein-coding genes can also promote efficient termination of Pol II transcription, and the results showed that this highly efficient pattern of termination is specific to tRNA genes (new analysis, Supplementary Figure 4a-h). As the reviewer suggested, we also analyzed the correlation between termination window size and intergenic distance, direction, and transcription level of downstream protein-coding and/or non-coding genes. The genome-wide analysis showed that the termination window size of genes arranged in tandem is similar to genes arranged in convergent direction (new analysis, Supplementary Figure 6a). We found that termination window size is slightly positively correlated with intergenic length and slightly negatively correlated with the transcription level of downstream genes (new analysis, Supplementary Figure 6b-d). We have updated the text accordingly in the revised manuscript to include these new results (L220) – “ We also analyzed the correlation between termination window size and intergenic distance, direction, and transcription level of downstream protein-coding and/or non-coding genes, respectively. The genome-wide analysis showed that the termination window size of genes arranged in tandem is similar to genes arranged in convergent direction (Fig S6a). We found that termination window size is slightly positively correlated with intergenic length and slightly negatively correlated with the transcription level of downstream genes (Fig S6b-d). ”*

3. Line 199 The xrn3 experiment, the author sequenced the nuclear RNA instead of chromatin bond RNA to capture more 3' cleavage products. Is there any evidence supporting this statement?

*Reply: We thank the reviewer for this important comment, and our apology for didn’t explain it clearly in the text. Our reasoning for using the nuclear fraction in the xrn3 experiment was that we were not sure if the 5’ or 3’ cleavage products are closely bound to the chromatin as the elongating RNAs do. It turned out that both the nuclear fraction and the chromatin-bound fraction can efficiently capture the cleaved and readthrough transcripts (new analysis, Supplementary Figure 7a). Since nuclear RNA isolation involves fewer steps than chromatin-bound RNA isolation, we have chosen nuclear RNA to make all the new FLEPseq libraries (please see updated Methods). We have revised the manuscript to clarify the reasoning of using nuclear RNA as input for FLEP-seq (L244) –*

*“It turned out that both the nuclear fraction and the chromatin-bound fraction can efficiently capture the cleaved and readthrough transcripts (Fig. S7a). Since nuclear RNA isolation involves fewer steps than chromatin-bound RNA isolation, we proceeded with nuclear RNA to make FLEP-seq libraries in a series of mutants and wildtype controls (please see Methods for detail).”*

4. Line 223 The accumulation of cleaved readthrough transcription which are most likely processed by the cleavage and polyadenylation complex in mutant xrn3 has been proved in previous studies (reference 12). In addition, the author concluded that some cleaved readthrough transcription can yield spliced and polyadenylation mRNA without their own transcription initiation. However, according to the examples in Fig. 6, nearly all spliced transcripts are separated from the upstream gene. Actually, it is hard to distinguish whether these transcripts are the cleaved products of upstream transcriptional readthrough or the products of new initiation. Are there biological replicates for both wildtype and xrn3 plants? More evidence, such as manual verification of certain loci by RT-PCR/seq at both nascent RNA and mRNA levels should be provided. Thus, the conclusion is not rigorous enough.

*Reply: We thank the reviewer for this important comment. To better distinguish the cleaved products and upstream transcriptional readthrough, we have added a zoomed-in view around the TSS site of AT1G73530 in the main figure, which clearly showed the difference of polyadenylated transcripts between wildtype and the atxrn3 mutant (new analysis, Figure 6b). We also reordered the reads at AT1G76180-AT1G76170 region for a clearer demonstration of their origin, which showed that most reads of the downstream AT1G76170 come from the cleaved readthrough of the upstream gene AT1G76180 in atxrn3 mutant(new analysis, Supplementary Figure 11).*

*As the reviewer suggested, we also constructed two new FLEP-seq libraries from the biological replicates of both the wildtype and the atxrn3 mutant (new data). The results from the two replicates are highly consistent in showing the transcripts of AT1g73530 in atxrn3 mostly originate from the upstream poly(A) site as cleaved products of upstream transcriptional readthrough, instead of their own initiation (new data, Figure 6b).*

*Regarding verification at the mRNA level, we have analyzed previously published RNA-seq data for both wide-type and atxrn3 mutant (Krzyszton et al., Plant J., 2018). The coverage plot confirmed that many more reads are aligned to the intergenic region of AT1G73540-AT1G73510 in atxrn3 mutant, compared to the few reads at the same regions in wildtype (new analysis, Figure 6c). We have added these important new updates to the main text (L291) –*

*“ This can be clearly seen with a zoomed-in view around the TSS site of AT1G73530 in two biological replicates of the wildtype and atxrn3 FLEP-seq libraries, showing that some polyadenylated transcripts of AT1G73530 in atxrn3 originated from the upstream poly(A) site as cleavage products of upstream transcriptional readthrough, instead of their own initiation (Fig. 6b). A reordered view of reads at AT1G76180-AT1G76170 region also confirms this observation, with most reads of the downstream AT1G76170 come from the cleaved readthrough of the upstream gene AT1G76180 (Fig. S11). Besides nascent RNA, we also check the mRNA level by analyzing previously published RNA-seq data of wide-type and atxrn3 mutant [14]. The coverage plot confirmed that more reads are aligned to the intergenic region of AT1G73540-AT1G73530 in atxrn3 mutant, compared to the fewer reads at the same regions in wildtype (Fig. 6c).”*

5. For Figure4-6, other than the certain gene/locus cases, genome wide analysis is expected to make a general point of view. How many genes' termination is regulated by the downstream tRNA or other type of genes? How many genes' termination is xrn3 sensitive and what is the potential determinants? How many genes' cleaved readthrough transcripts enter the downstream genes in xrn3?

*Reply: We thank the reviewer for this important suggestion. For the 34 genes whose termination is regulated by the downstream tRNA in tandem direction, 26 of them have termination window ends located approximately 60 nt (55~65 nt) upstream of the tRNAs. We have also checked if snRNA, snoRNA, and protein-coding genes can also promote efficient termination of Pol II transcription, and the results showed that this highly efficient pattern of termination is specific to tRNA genes (new analysis, Supplementary Figure 4a-h).*

*Regarding the genome-wide analysis of termination in atxrn3, we compared the size of termination window in the atxrn3 and wildtype, and the result showed a strong impact of atxrn3 on termination window size at hundreds of gene loci (new analysis, updated Figure 5d). The termination window of 354 genes were statistically longer in the atxrn3 mutant than in wildtype (Mann–Whitney U-test, p value < 0.001). We identified 14 genes with cleaved readthrough transcripts entering their immediate downstream genes (read counts ≥ 15) in atxrn3 mutant (new analysis, Supplementary Table 3).*

*As to what are the potential determinants that make these genes atxrn3 sensitive, this is an excellent question by the reviewer that we don’t have a clear answer yet. It is worth noting that the atxrn3 alleles we and other previous studies sequenced are weak alleles, and the strong allele of atxrn3 is embryo lethal (Gy et al., The Plant Cell, 2007), which suggests AtXRN3 may have a much stronger impact than revealed from the weak alleles. We hope future works from other groups and us would be able to gradually address how AtXRN3 recognizes its targets.*

*We thank the reviewer for the insightful comments and we have revised manuscript at multiple places to include these new points –*

*“ For the 34 genes whose termination is regulated by the downstream tRNA in tandem direction, 26 of them have termination window ends located ~ 60 nt upstream of the tRNAs. ”(L198)*

*“ From a genome-wide perspective, we compared the size of termination windows in the atxrn3 mutant and in wildtype, and the result showed a strong impact of atxrn3 on termination window size at hundreds of gene loci (Fig. 5d). The termination window of 354 genes were statistically longer in the atxrn3 mutant than in wildtype (Mann–Whitney U-test, p value < 0.001). ” (L260)*

*“ Furthermore, we identified 14 genes with cleaved readthrough transcripts entering their immediate downstream genes in atxrn3 mutant (Table S3). ”(L282)*

*“Another important question is what are the potential determinants that make hundreds of genes sensitive to atxrn3 mutation. We don’t have a clear answer yet, but it is worth noting that the atxrn3 alleles we and other previous studies sequenced are weak alleles, and the strong allele of atxrn3 is embryo lethal [47], which suggests AtXRN3 may have a much stronger impact than revealed from the weak alleles. ”(L363)*

Minor concerns,

1. Line 84, "Taking DRM2 as an example"

*Reply: We thank the reviewer for catching this error. We have corrected it in the revision.*

2.Line 90, pNET-seq and plaNET-seq sequence NRPB1 and NRPB2 associate RNA respectively. The pNET-seq is capable to detect different RNA Pol II isoform and thus may distinguish Pol II at certain transcription step.

Reply: We thank the reviewer for this important point and our apology for the insufficient introduction. We revised the text to expand the introduction on pNET-seq (L97) –

*“ Previous short-read based nascent RNA sequencing methods, such as pNET-seq, plaNET-seq and GROseq, have been applied in Arabidopsis [41–44] and in other plants including cassava and maize [45], are mostly developed for detecting Pol II-associated elongating RNAs, and can also detect RNA signal downstream of poly(A) site (Fig. 1b, lower panel). In particular, the pNET-seq method can detect nascent RNAs that are associated with various isoforms of Pol II CTD, including total CTD, unphosphorylated, phosphorylation on Ser 2 and Ser 5 [43], thus may distinguish Pol II at certain transcription step. ”*

3.Figure 1b, AT2G3830 should be in italics.

*Reply: We thank the reviewer for catching this error. We have corrected it in the revision.*

4.Figure 1b, in the pNET-seq track, which antibody was used for this experiment? Ser2P Pol II is supposed to detect the nascent RNA post PAS.

*Reply: We apologize for not mentioning this important information. According to the original paper (Zhu et al., Nature Plants, 2018), the authors used antibody CMA602 to capture Pol II Ser2P in chromatin. We have updated the Pol II Ser2P pNET-seq data in Figure 1b (new analysis, updated Figure 1b). We also added the detailed description in the figure legend in the revision.*

5.Figure 2c, d. Why is the number of genes different? It should be explained in figure legend.

*Reply: We apologize for the confusion. We require a minimum of 15 readthrough reads for calculating the termination windows, which leads to different number of genes qualified for analysis among the two replicates and pooled samples. We have added an explanation about the different numbers in the figure legend.*

6.Figure3, it will be helpful to compare FLEP-seq with Ser2P and Ser5P-seq data.

*Reply: We thank the reviewers for this suggestion. We have added a new panel in Figure 3 comparing the coverage of FLEP-seq and pNET-seq with Ser2P and Ser5P at the TWE sites (new analysis, Figure 3b). We have added this result in the main text (L166) – “The presence of termination window engaged Pol II peak could also be observed in pNET-seq [43] and GRO-seq [42] data (Fig. 3b, Fig. S3a).”*

7.Figure 5c. The gene name in the brackets should be in italics.

*Reply: We thank the reviewer for catching this error. We have corrected it in the revision.*

8.Figure 5b. Why there is no sharp peak enriched at pA site for WT as presented in Fig.1f.

*Reply: We apologize for the confusion. The previous Figure 5b is the plot for the 5’ end of the reads, while Figure 1f is for the 3’ end. Because AtXRN3 is a 5’->3’ exonuclease, the peak of 5’ end of the cleaved RNA only accumulates in the atxrn3 mutant. We have changed the title of the panel to “5’ end (not 3’) distribution” to make this point clearer (Updated Figure 5a). We also highlighted its difference with the panel in Figure 5a and in the legends of Figure 5.*

9.Figure 6a, b. The border lines are inconsistent.

*Reply: We thank the reviewer for catching this error. We have corrected it in the revision.*

10.Line 267. As a scientific name of a species, Arabidopsis thaliana should be in italics.

*Reply: We thank the reviewer for catching this error. We have corrected it in the revision.*

**Second round of review**

**Reviewer 1**

Thank authors to add more data to support their claims. Generally, the answers are acceptable, however, still some answers are not inaccurate which may mislead readers.

(1) for 4tU-Seq, it is only a method to label nascent RNAs, the sequencing can be done with illumina or FLEP-Seq or other sequencing methods, e.g. pacbio-seq, etc., therefore, 4tU-seq is not specific for short-read sequencing. Actually, authors also cited the molecular cell paper (Drexler et al., Molecular Cell, 2020) to show that nascent RNA (4sU-seq)-seq also can be done with ONT DRS.

(2) although standard ONT DRS kit is designed for poly(A) RNAs, customer-specific RT adaptor also work well, which has been confirmed for 16S rRNA sequencing.

(3) met1-1 is not a knock-out line, it still remain 25% (75% loss) CG methylation according to the literature the authors cited, while met1-3 is a knock-out line, and it nearly lost all CG methylation, authors cannot use met1-1 to claim the DNA methylation effects on poly(A) site selection and related transcriptional termination events.

(4) authors performed BS-seq for atxrn3 mutant, but authors only compare CG methylation, not consider CHG and CHH methylation effects. Actually author already showed that GC content in the regions is lower, why authors did not show the effects of CHG and CHH methylation. Li. et al. actaully have observed that DNA methylation has effects on poly(A) site selection (https://www.biorxiv.org/content/10.1101/2021.04.29.441938v1.full), authors should also show the results of CHG and CHH methylation.

**Reviewer 2**

In this revision, the authors of "Landscape of Transcription Termination in Arabidopsis Revealed by Single-molecule Nascent RNA Sequencing" have included two more FLEP-seq experiments for two mutants met1 and fpa, and one additional replicate of atxrn2, which support that FLEP-seq is a useful tool to study transcription termination especially for plant genes with short termination windows. They did chromatin status analyses in order to explore the mechanism of termination and transcription readthrough. Although most results from bioinformatic analyses are negative, it is meaningful for further transcription termination studies. They also integrated their data in a genome browser for the easily visiting. They addressed all my comments except for the bdr and CPSF mutants, which is OK. Taken together, the revision is substantially improved. However, I still have some concerns, Line 197, “Our results showed that this highly efficient pattern of termination is specific to tRNA genes”. Did the authors mean “Our results showed that this highly efficient pattern of termination is specific to genes to which a tRNA gene locates downstream”? Line 345-348, the short read sequencing may distinguish the 5’ and 3’ of each RNA molecule using pair-end sequencing method, which is popular at the moment. There are several protocols determining both ends, such as TIF-seq. Line 349, the authors argue that the single molecule sequencing/long reads sequencing is less vulnerable than short read sequencing in determining the termination windows. Given that the 3’ end position of each nascent/chromatin bound/nuclear RNA was used in determine the termination windows, both long and short read sequencing are sensitive to the sequencing depth to the same degree. In the term, short read sequencing is more advantageous because of its deeper sequencing yields. In the discussion section, the authors try to compare the FLEP-seq with ONT DRS. For the audience who are not familiar with nanopore or third generation sequencing, it would be helpful to add a brief introduction of ONT DRS.

**Authors Response**

**Point-by-point responses to the reviewers’ comments:**

Reviewer #1:

Thank authors to add more data to support their claims. Generally, the answers are acceptable, however, still some answers are not inaccurate which may mislead readers.

*Reply: We appreciate the constructive comments from the reviewer that helped us to substantially improve the manuscript.*

(1) for 4tU-Seq, it is only a method to label nascent RNAs, the sequencing can be done with illumina or FLEP-Seq or other sequencing methods, e.g. pacbio-seq, etc., therefore, 4tU-seq is not specific for short-read sequencing. Actually, authors also cited the molecular cell paper (Drexler et al., Molecular Cell, 2020) to show that nascent RNA (4sU-seq)-seq also can be done with ONT DRS.

*Reply: We agree with the reviewer that metabolic-labeling experiments, such as 4tU-seq and 4sU-seq, are the method to detection newly synthesized RNA (Wissink et al., Nature Reviews Genetics, 2019), and can also combined with long-read sequencing to capture full-length nascent RNA. We have updated this information in the discussion part (page 17).*

*“Metabolic-labeling based methods, such as 4tU-seq and 4sU-seq, can detect newly synthesized RNA, and can be combined with long-read sequencing platforms to characterize full-length nascent RNA [34].”*

(2) although standard ONT DRS kit is designed for poly(A) RNAs, customer-specific RT adaptor also work well, which has been confirmed for 16S rRNA sequencing.

*Reply: We thank the reviewers for the comment. We have added the new text to the discussion part accordingly (page 18).*

*“The current ONT DRS kit is designed for profiling polyA+ RNAs, additional steps such as in vitro polyadenylation [33, 34] or using customer-specific RT adaptor [70, 71] can be applied to non-polyA RNAs.”*

(3) met1-1 is not a knock-out line, it still remain 25% (75% loss) CG methylation according to the literature the authors cited, while met1-3 is a knock-out line, and it nearly lost all CG methylation, authors cannot use met1-1 to claim the DNA methylation effects on poly(A) site selection and related transcriptional termination events.

*Reply: We thank the reviewer for this very insightful comment. The met1-1 mutant is indeed a weaker allele compared to met1-3, but the remaining DNA methylation in met1-1 is mostly at TE regions, and the genic regions lost almost all CG methylation as reviewed by WGBS of met1-1 (new data, Figure S13a, b). Nevertheless, we fully agree with the reviewer that the strong met1-3 allele is a better material to use, and recent work from Li et al. has already nicely explored the relationship between DNA methylation and poly(A) site selection. Therefore, we have cited the Li et al. paper and deleted previous claims regarding DNA methylation and poly(A) site selection (page 14).*

*“However, it is worth noting that met1-1 is not a null allele and still have some remaining CG methylation at TE regions (Fig. S13b), recent characterization using ONT direct RNA sequencing of full-length mRNA from the strong met1-3 allele, which removes virtually all CG methylation, has discovered the effects of DNA methylation on splicing site and poly(A) site selection, as well as on poly(A) tail length [66].”*

(4) authors performed BS-seq for atxrn3 mutant, but authors only compare CG methylation, not consider CHG and CHH methylation effects. Actually author already showed that GC content in the regions is lower, why authors did not show the effects of CHG and CHH methylation. Li. et al. actually have observed that DNA methylation has effects on poly(A) site selection (https://www.biorxiv.org/content/10.1101/2021.04.29.441938v1.full), authors should also show the results of CHG and CHH methylation.

*Reply: We thank the reviewer for this suggestion. We have now added the result of CHG and CHH methylation (new figure, Figure S13c). Because the non-CG methylation levels at genic regions are low in general, we did not observe an obvious difference between WT and the atxrn3 mutant (Figure S13c). We thank the reviewer for pointing out the highly relevant Li et al. paper, and we have cited their discovery on the role of DNA methylation on poly(A) site selection. The Li et al. paper did a very nice analysis on identifying novel TTSs in various genotypes, and we have therefore deleted our weak statement drawn mostly on the comparison of known major and minor poly(A) sites, and we also removed the previous Fig. S12c and S12d accordingly (page 16).*

*“However, individual poly(A) sites could still be affected, as a recent work has discovered large number of novel TTSs in the met1-3 mutant, suggesting a role of DNA methylation in poly(A) site selection [66].”*

Reviewer #2:

In this revision, the authors of "Landscape of Transcription Termination in Arabidopsis Revealed by Single-molecule Nascent RNA Sequencing" have included two more FLEP-seq experiments for two mutants met1 and fpa, and one additional replicate of atxrn2, which support that FLEP-seq is a useful tool to study transcription termination especially for plant genes with short termination windows. They did chromatin status analyses in order to explore the mechanism of termination and transcription readthrough. Although most results from bioinformatic analyses are negative, it is meaningful for further transcription termination studies. They also integrated their data in a genome browser for the easily visiting. They addressed all my comments except for the bdr and CPSF mutants, which is OK. Taken together, the revision is substantially improved. However, I still have some concerns,

*Reply: We appreciate the helpful and constructive comments from the reviewer.*

Line 197, “Our results showed that this highly efficient pattern of termination is specific to tRNA genes”. Did the authors mean “Our results showed that this highly efficient pattern of termination is specific to genes to which a tRNA gene locates downstream”?

*Reply: We thank the reviewer for catching these errors. We have revised the sentence accordingly.*

Line 345-348, the short read sequencing may distinguish the 5’ and 3’ of each RNA molecule using pair-end sequencing method, which is popular at the moment. There are several protocols determining both ends, such as TIF-seq.

*Reply: We thank the reviewer for the comments. We have added the description of TIF-seq and its usage of paired-end sequencing to capture both the 5’ and 3’ end of each RNA molecule (page 17).*

Line 349, the authors argue that the single molecule sequencing/long reads sequencing is less vulnerable than short read sequencing in determining the termination windows. Given that the 3’ end position of each nascent/chromatin bound/nuclear RNA was used in determine the termination windows, both long and short read sequencing are sensitive to the sequencing depth to the same degree. In the term, short read sequencing is more advantageous because of its deeper sequencing yields. In the discussion section, the authors try to compare the FLEP-seq with ONT DRS. For the audience who are not familiar with nanopore or third generation sequencing, it would be helpful to add a brief introduction of ONT DRS.

*Reply: We thanks the reviewer for these insightful comments. We have updated the discussion to include the advantages of using short-read sequencing to measure termination window (page 17), as well as a brief introduction of ONT DRS when it is first introduced (page 4).*
